# Synthesis and functionalization of polymeric materials based on organic borazine

**DOI:** 10.1039/d5ra04671h

**Published:** 2025-09-02

**Authors:** Xuewei Fu, Jiating Feng, Ziyu Wang, Huanfeng Jiang, X. Fu, J. Feng, Z. Wang, H. Jiang

**Affiliations:** a Key Laboratory of Functional Molecular Engineering of Guangdong Province, School of Chemistry and Chemical Engineering, South China University of Technology Guangzhou P. R. China jianghf@scut.edu.cn

## Abstract

Borazine is also known as “inorganic benzene”. Because of their unique boron–nitrogen (B–N) isoelectronic structure, high thermal, chemical stability, and tunable electronic properties, borazine polymers are green candidates to replace traditional halogen-containing or hazardous polymers. This review describes the synthesis of borazine and its derivatives and their applications in the functionalization of polymeric materials, recent advances, directions of research, and potential for future development in various fields. The performance of borazine-based polymeric materials from thermal, mechanical, optical, electrical, catalytic and adsorption aspects is highlighted. Their potential applications in aerospace, electronics, energy, environment and other fields are also summarized.

## Abstract 2

This work summarizes the latest breakthroughs in borazine synthesis methods, precursor design, and hybrid materials. It provides insights and directions for translating these theoretical potentials into practical technological innovations.

## Graphical abstract

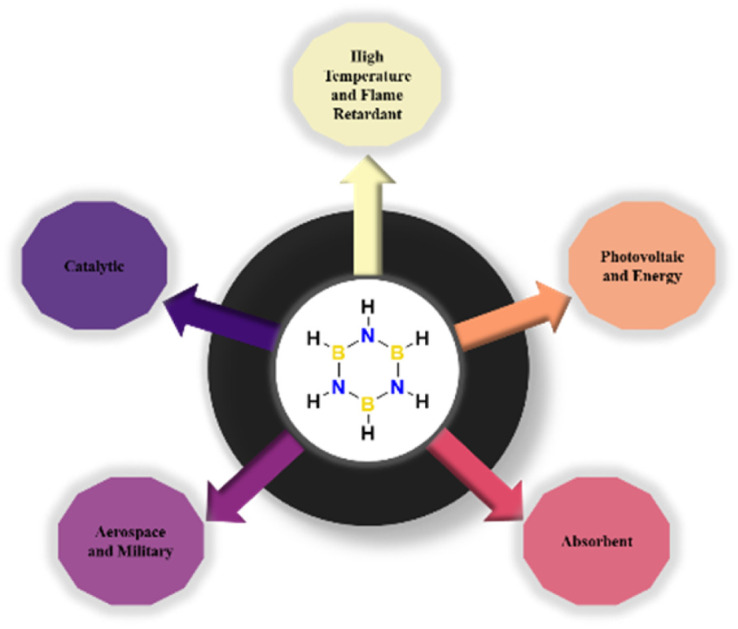

## 1. Introduction

**Fig. 1:**
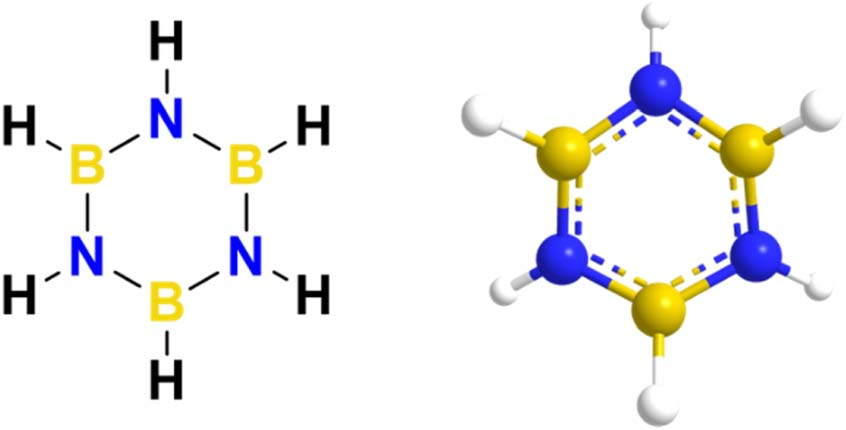
Molecular and 3D microstructure of borazine.

Boron and nitrogen compounds, as analogues of carbon materials, have received considerable attention due to their unique physicochemical properties. As representatives of boron and nitrogen compounds, cyclic borazines (borazine) and their derivatives show great potential for application in the field of functional polymer materials due to their special electronic structure and reactivity. Borazine (B_3_N_3_H_6_) is a planar six-membered ring consisting of alternating B–N bonds with bond lengths (B–N about 1.44 Å) intermediate between single and double bonds, exhibiting partial conjugation properties.^1,2^ An inorganic heterocyclic compound with a six-membered aromatic ring structure, it consists of three boron atoms alternating with three nitrogen atoms, as shown in Fig. 1.^3–5^ It contains either inorganic or organic groups that are isoelectronic with benzene and therefore have similar physical properties.^6,7^ However, due to the different electronegativities of the boron and nitrogen atoms in the molecule, its unique electronic structure, characterized by partial delocalization of π-electrons, confers distinctive chemical and physical properties.^8^

Due to the polarity of the B–N bond, borazine exhibits strong UV absorption and emission properties, and has electrical insulation properties.^9–11^ These properties make borazine an ideal dopant for modulating the optoelectronic properties of carbon-based materials. The incorporation of borazine units into the polymer backbone or side chains can give the materials many excellent properties, such as excellent thermal stability, low dielectric constant, excellent optical properties and significant gas adsorption ability. These properties make borazine an attractive building block for the development of advanced polymeric materials.^12–20^

**Fig. 2:**
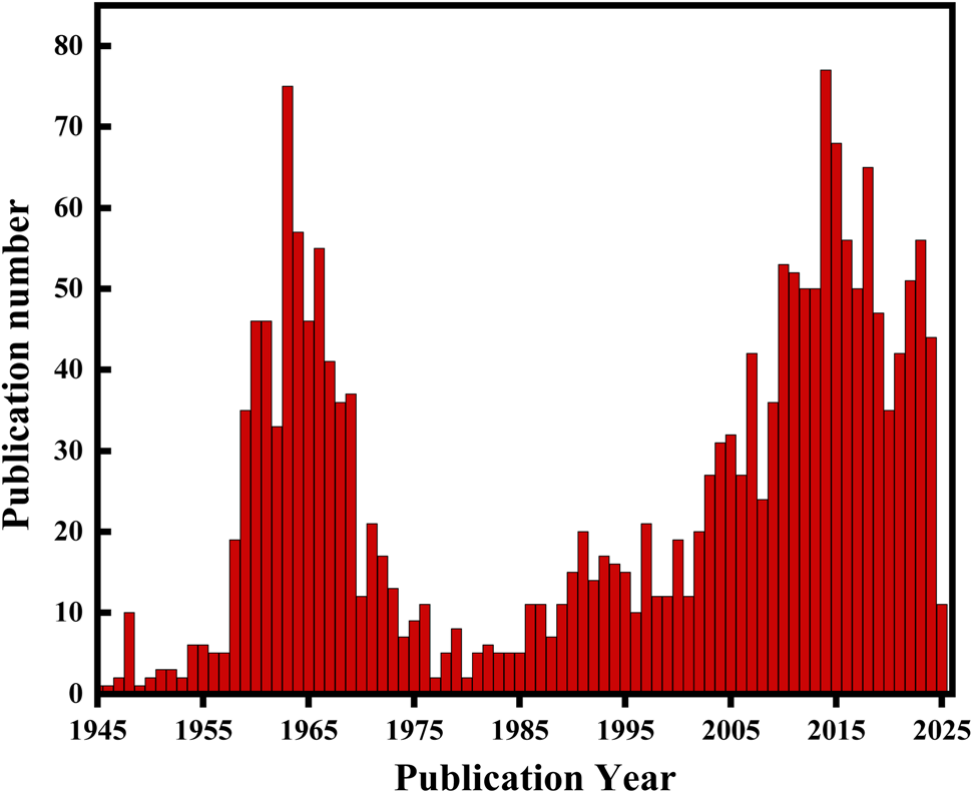
Annual publication statistics on borazine and its derivatives from 1945 to 2025 (data sourced from SciFinder).

Between 1960 and 1970, a series of publications provided the first comprehensive investigations of the reactivity of borazine and laid the foundation for the subsequent exploration of the chemical properties and reaction mechanisms of borazine and its derivatives.^21–23^ Borazine and its derivatives, particularly polycyclic aromatic compounds doped with boron–nitrogen elements, have been widely applied in fields such as ultraviolet emission, ceramic materials and hydrogen storage materials due to their distinct physicochemical properties.^24,25^Fig. 2 shows the publication volume of borazine and its derivatives from 1945 to 2025. It can be seen that these compounds have gradually attracted widespread attention from researchers.

There have been a number of recent advances in the study of borazine-based polymeric materials.^26–29^ In terms of synthesis, researchers have developed a variety of new borazine monomers and polymerization methods, mainly as follows:

### 1.1 Covalent bond functionalization

Covalently bonded polymer networks are constructed by reacting the boron or nitrogen atoms of borazine with the reactive groups (*e.g.*, hydroxyl groups, amino groups) of polymer chains.^30–32^ This includes the use of boron atoms of borazine to react with *o*-diols to form dynamically reversible borate bonds that give the materials self-healing capabilities.^33–36^ Copolymerization of borazine derivatives containing reactive groups with other monomers. Bonifazi synthesized a series of hexaaryl-substituted borazine derivatives and prepared borazine-doped polycyclic aromatic hydrocarbons (PAHs) by planarization of the borazine ring *via* an intramolecular Friedel–Crafts reaction.^37–39^ This provides a new idea for the construction of borazine-based conjugated systems. Additionally, there is aziridine ring-opening polymerization,^40–42^ which uses borazine as a monomer to create polymers with B–N backbones, like polyborazine (PBN).^43–45^ Direct polymerization using borazine derivatives as monomers. For example, borazine-based polymers can be made by polymerizing trichlorocycloborazane (TCB) by reacting it with other compounds.^46^ Alternatively, borazine-based ceramic materials have been prepared by pyrolysis using borazine as precursors. Liu *et al.* prepared Si_3_N_4_–BN composites by an impregnation-cracking process using borazine as a precursor, which showed good mechanical properties.^47^

### 1.2 Noncovalent functionalization

Cycloboronazane is introduced into the polymer matrix by hydrogen bonding, π–π stacking or electrostatic interaction.^19,29,48^ Borazine modification of existing polymers. For example, the borazine structure is introduced by reacting a polycarbosilane with trichlorocycloborazane.^49^ These synthetic methods provide a variety of options for the design and preparation of structurally controllable borazine-based polymeric materials.

Borazine-based polymer materials have many unique properties and applications, and the unique properties of borazine-based polymers have led to their development in a variety of applications. Borazine-based polymers exhibit some gas adsorption properties and can be used for gas storage and separation.^50–56^ Due to the introduction of the borazine ring, the materials have excellent oxidation resistance and thermal stability and can be used for high-temperature applications.^57–59^ Borazine-based polymers typically have low dielectric constants and dielectric losses and can be used in electromagnetic wave transparent materials and microelectronic packaging. It is also possible to modulate the electronic structure of the materials to develop new functionalized materials with excellent optoelectronic functions.^58,60–62^ In terms of performance, borazine-based polymers exhibit a wide range of excellent properties. Jackson's team produced a series of porous borazine-conjugated polymers that exhibited excellent hydrogen and carbon dioxide adsorption.^63^ Kim's team produced highly thermally conductive composites by surface-modifying borazine nanosheets, significantly improving the thermal conductivity of the materials.^64,65^ Guo's team synthesized arylene resins with controllable amounts of borazine and found that the introduction of borazine effectively reduced the dielectric constant and dielectric loss of the materials.^66^ These results demonstrate the potential of borazine-based polymers for applications in gas storage, thermal management and electromagnetic shielding. At the application development level, borazine-based polymeric materials have shown promise in a number of areas. In the field of gas storage, the halogen-modified borazine-linked polymers prepared by Reich and Jackson have shown excellent CO_2_/CH_4_ selective adsorption performance.^67^ In the field of wave-transparent materials, Cao's team has prepared boron nitride-based wave-transparent composites using silicon nitride–oxygen fibers as reinforcement and borazine as a precursor, which are suitable for hypersonic vehicle antennae.^68^ In the field of microelectronic packaging, the borazatruxene molecules synthesized by Limberti's team have a unique structure that combines an electron-conducting region with an electron-insulating region, which is expected to be an important material for molecular electronic devices.^69^ In terms of processing, precursor methods allow the production of borazine-based ceramics and composites. Polyborazine precursors derived from borazine can be converted to boron nitride ceramics after pyrolysis.^47,50^ This allows the preparation of boron nitride fibers, films and porous structures. Composites combining boron nitride compounds with carbon nanotubes or other fillers have been developed to achieve higher thermal and mechanical properties. These studies clearly show that borazine-based polymer materials have a wide range of applications in energy and environment, aerospace, electronics and information.

By precisely controlling the reactivity of the borazine ring, bio-based monomer derivatization, low-toxicity functionalization, and the incorporation of self-repairing features can be accomplished, considerably increasing the material's sustainability throughout its life cycle. The current problems are focused on large-scale green preparation, long-term stability in complicated contexts, and recycling cost control, with the goal of breaking through the bottleneck of balancing performance with environmental advantages. Therefore, in this article, we first outline the structure and properties of borazine, systematically summarize the synthesis and focus on typical cases of polymers prepared using borazine and its derivatives as monomers or cross-linking agents. Then, we systematically discuss and summarize the research progress on the application of borazine-based polymers in various fields. Their potential applications in areas such as high-temperature insulators, gas adsorption and separation, optoelectronic materials, catalysts, and aerospace are highlighted, and future directions are discussed.

## 2. Structure and properties of borazine

Borazine (B_3_N_3_H_6_) is a six-membered cyclic compound consisting of boron (B), nitrogen (N) and hydrogen (H). The presence of conjugated π bonds in the borazine molecule has been confirmed by single-crystal X-ray diffraction analysis and its structure is very similar to that of benzene (C_6_H_6_). However, it exhibits significantly different chemical and physical properties due to its unique B–N bonding properties and differences in electron distribution.^1,2,6^

### 2.1 Geometrical structure and bonding characteristics

The molecular structure of cyclic boron aziridine shows a planar six-membered ring skeleton structure with six atoms (3 B and 3 N) arranged in alternating rows, and each B and N atom is connected by a B–N single bond. The B–N bond length is about 1.44 Å, which is intermediate between a single bond and a double bond, with the theoretical value of the B–N single bond being about 1.58 Å and the B=N being about 1.32 Å. This suggests that there is a partially double-bonding property, *i.e.*, out-of-domain π-bonding. The intra-ring B–N–B and N–B–N bond angles are close to 120°, which is consistent with that of the benzene ring and supports its planar conjugated structure.^70,71^

The electronic structure of borazine is electron deficient due to the fact that the boron atom has only six electrons in its outer layer, while the nitrogen atom has a lone pair of electrons, resulting in an electron-deficient ring as a whole. This property allows borazine to act readily as a Lewis acid and to react with electron-rich substances such as amines and ethers in coordination or charge transfer reactions. Unlike the six π-electrons of benzene, the π-system of borazine consists of three p-orbitals of B and three p-orbitals of N, forming an off-domain π-bond with six centers and six electrons, but the degree of off-domain is weaker than in the benzene ring due to the unequal distribution of electrons resulting from the differences in electronegativity of B and N.^72^

### 2.2 Isomerization and substituent effects

Substituent position isomerization of the neighbor (1,2-), interstitial (1,3-), and para (1,4-) isomers can occur when the H on the borazine is replaced by other groups (*e.g.*, alkyl, aryl, halogen). For example, when the substituent group is attached to the B atom, it is susceptible to attack by nucleophilic reagents due to the lack of electrons in B. When the substituent group is attached to the N atom, the lone pair of electrons is involved in the delocalization, which can affect the electronic properties of the ring.^73^ Meanwhile, the effect of substituents on the properties, electron-withdrawing groups such as –F and –NO_2_, exacerbate the electron-deficient nature of the ring and increase its ability to act as an electron acceptor. Electron-donating groups such as –NH_2_ and –OCH_3_ partially compensate for the electron-deficient nature of B and can modulate reactivity.^74^

### 2.3 Stability and reactivity of borazine

The high B–N bond energy, about 390 kJ mol^−1^, confers excellent thermal stability to borazine, which decomposes at temperatures above 300 °C. However, its thermal stability is lower than that of boron nitride (BN), which can form ceramic materials at high temperatures. Borazine is hydrolysis sensitive, due to the polarity of the B–N bond, it reacts easily with water to form boric acid (H_3_BO_3_) and ammonia (NH_3_).

It oxidizes slowly in air and needs to be stored in an inert atmosphere. The unique structure of cyclic boron nitrogen alkanes (planar six-membered ring, B–N partially excluded bond, electron deficient) is the core foundation for their functionalized applications. By precisely controlling the type and position of substituents, polymer materials with high stability, modifiability and multifunctionality can be designed to provide innovative solutions in the fields of optoelectronics, energy and biomedicine.

## 3. Functionalization reactions of borazine

The functionalization reactions of borazine are an important area of research in organic chemistry, involving the introduction of various functional groups on the boron–nitrogen ring to synthesize organic compounds with specific functions. The following is an introduction to the functionalization reactions of borazine.

**Scheme 1:**
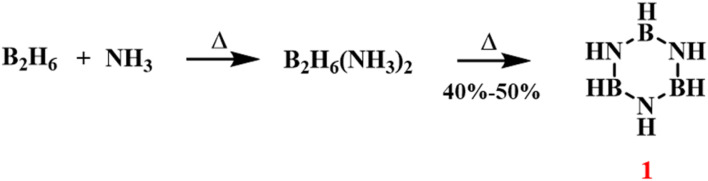
Synthesis of borazine through the thermal decomposition of NH_3_ and B_2_H_6_.^75^

In 1926, Stock synthesized borazine through a thermal decomposition reaction, combining ammonia (NH_3_) and diborane (B_2_H_6_) (Scheme 1).^75^ The reaction proceeds through the initial formation of a liquid adduct B_2_H_6_·NH_3_, which upon thermal activation undergoes structural rearrangement to yield the cyclic borazine framework with concomitant H_2_ evolution. The process exhibited moderate yields of 40–50%, with significant dependence on reaction parameters including temperature gradients, pressure regimes, and stoichiometric ratios. This process demonstrated that the synthesis of borazine could be driven by high-temperature treatment of boranes and ammonia to promote the formation of the cyclic structure. Single-crystal X-ray diffraction analysis revealed that B_3_N_3_H_6_ had a structure similar to benzene, with delocalized π bonds. Although the synthesis of borazine had been confirmed, the reaction yield could fluctuate under different experimental conditions, and achieving high yield and purity remained a challenge.

**Scheme 2:**
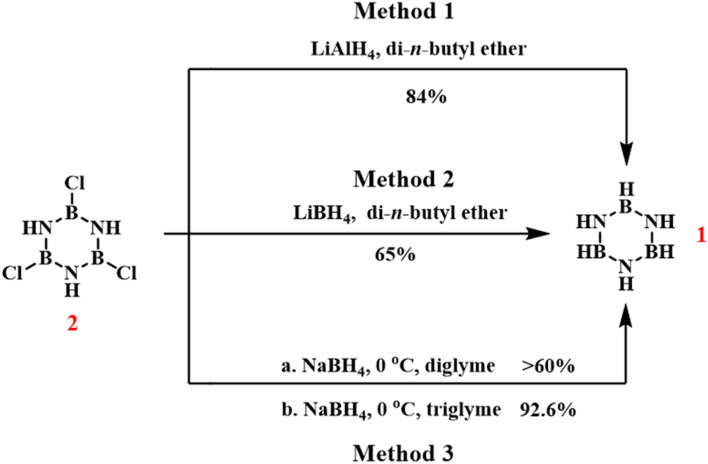
Routes for the reduction of *B*-trichloroborazine to borazine.^76,77^

In 1954, Schaeffer and colleagues reduced *B*-trichloroborazine to borazine (Scheme 2, methods 1 and 2) using lithium borohydride (LiBH_4_) and lithium aluminum hydride (LiAlH_4_).^76^ Using di-*n*-butyl ether as the solvent, LiAlH_4_ reacted with *B*-trichloroborazine, yielding borazine with an 84% yield. However, the presence of the byproduct AlH_3_ made it difficult to recover borazine, and large-scale reactions could not be performed. In the same system, reducing *B*-trichloroborazine with LiBH_4_ avoided the separation difficulties associated with AlH_3_, yielding borazine with a 65% yield. The generated B_2_H_4_ could then react with NaBH(OCH_3_)_3_ to form NaBH_4_.

Six years later, Hohnstedt and Haworth proposed that since B_2_H_5_ can react with NaBH(OCH_3_)_3_ to regenerate NaBH_4_, it would also be reasonable and advantageous to use NaBH_4_ to reduce *B*-trichloroborazine to borazine (Scheme 2, method 3).^77^ When implementing this approach at 0 °C in diglyme, borazine was obtained above 60% yield. Remarkably, solvent optimization using triglyme significantly enhanced the yield to 92.6%.

**Scheme 3:**
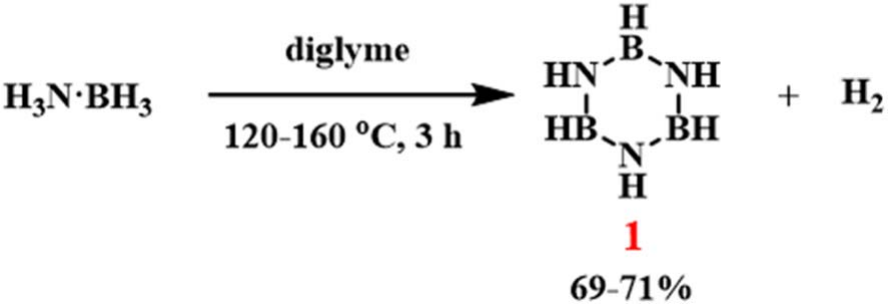
Thermal decomposition of ammonia borane to form borazine.^78^

In 1995, Gallery Chemical Co. developed a method for the large-scale commercial synthesis of borazine (Scheme 3).^78^ They used ammonia borane dissolved in diglyme for batch pyrolysis, achieving a yield of 69–71% of borazine at scales ranging from 0.5 to 3 g. Subsequently, a more complex continuous flow process was adopted, utilizing a heated vertical steel reactor to produce borazine on a larger scale. However, this complex design proved difficult to implement in a routine laboratory setting.

**Scheme 4:**
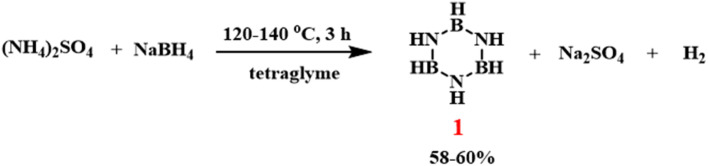
Synthesis of borazine by the reaction of (NH_4_)_2_SO_4_ and NaBH_4_.^79^

The method of preparing borazine by reducing *B*-trichloroborazine has several limitations—long reaction times, difficult purification, the use of potentially carcinogenic solvents (such as chlorobenzene), and the need to handle air-sensitive substances (*B*-trichloroborazine and B_2_H_5_). Additionally, the preparation procedure developed by Gallery Chemical Cooperation requires complex engineering equipment, making it unsuitable for laboratory-scale reactions. Based on these limitations, Sneddon developed a convenient procedure for the laboratory-scale preparation of borazine (Scheme 4).^79^ By dissolving (NH_4_)_2_SO_4_ and NaBH_4_ in tetraglyme and reacting them at 120–140 °C for 3 h, the borazine was obtained after vacuum distillation and purification (trapped at −78 °C), with a yield of 58–60%. This is a simple, fast method that allows the synthesis of several grams of borazine in a single step from inexpensive reagents using standard laboratory equipment.

**Scheme 5:**
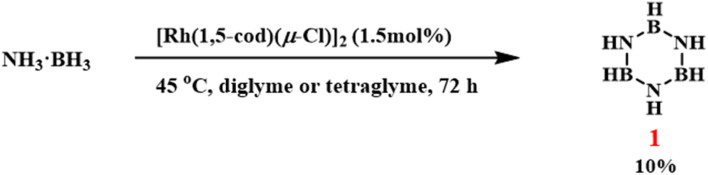
[Rh(1,5-cod)(μ-Cl)]_2_-catalyzed borazine synthesis.^80^

Traditional synthetic routes to borazine generally require elevated temperatures to activate the substrates. To improve reaction efficiency, researchers have explored various catalytic systems. In 2001, Manners and coworkers reported that 1.5 mol% of [Rh(1,5-cod)(μ-Cl)]_2_ catalyzed the dehydrogenative coupling of aminoborane to form borazine at 45 °C (Scheme 5).^80^ The study identified rhodium nanoparticles and metal clusters generated during the reaction as active catalytic species. Although this system exhibited good selectivity, it required a prolonged reaction time (72 h), faced challenges in product isolation *via* vacuum fractional distillation, and achieved a final yield of only 10%.

**Scheme 6:**
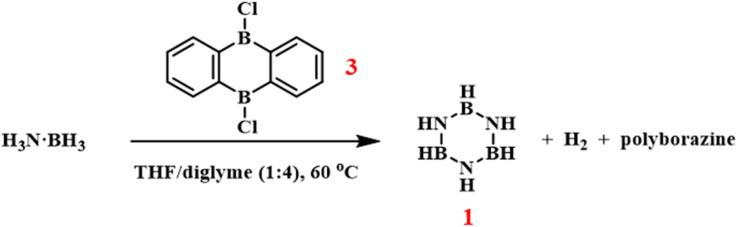
Dehydrogenative synthesis of borazine using 9,10-dichlorodibora-anthracene catalyst.^81^

Wegner *et al.* employed the Lewis acid 9,10-dichlorodiboraanthracene as a metal-free catalyst to promote dehydrogenative coupling of ammonia borane at 60 °C, forming borazine (Scheme 6).^81^ This approach eliminates environmental risks associated with transition-metal catalysts and exhibits notable catalyst reusability. However, as the study primarily aimed to develop metal-free systems for hydrogen release from ammonia borane, the borazine yield data were not reported in this work.

### 3.1 Hydrocarbylation reaction

#### 3.1.1 Alkylation reactions

**Scheme 7:**
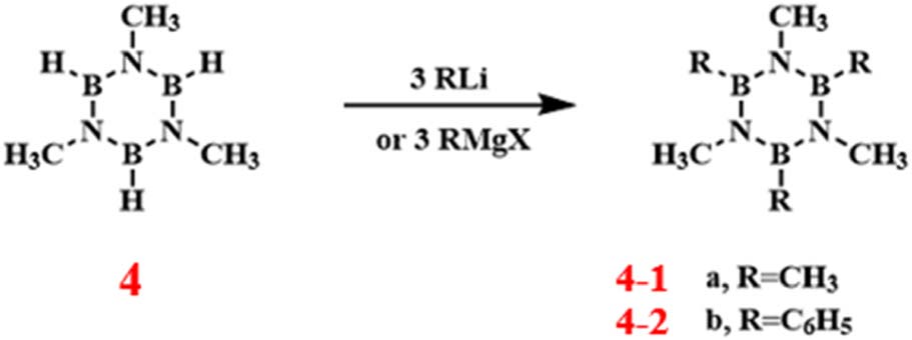
Synthesis route of *B*-alkylated *N*-trisubstituted borazine.^82^

In 1959, Janet Hall Smalley and others first reported the *B*-alkylation and *B*-arylation reactions of *N*-trisubstituted borazine using Grignard or lithium reagent to synthesize *B*-substituted borazine compounds (Scheme 7).^82^ The study revealed that there is a difference in the reactivity of 4-2 and 4-1 in *B*-substitution reactions, with the 4-2 being more reactive. This finding offers a new perspective for understanding the electronic structure and reactivity of borazines.

**Scheme 8:**
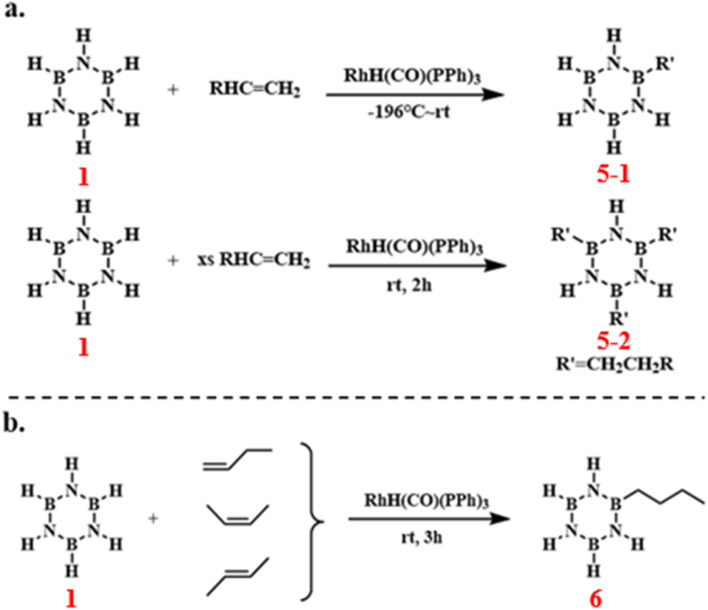
(a) Synthesis route of *B*-substituted monoalkyl, dialkyl, and trialkyl borazine;^83^ (b) Reaction of borazine with 1-butene, *cis*-2-butene, and *trans*-2-butene.^84^

In 1994, Paul J. Fazen and others introduced a novel transition metal-catalyzed approach to produce *B*-substituted monoalkyl, dialkyl, and trialkyl borazines efficiently and selectively under mild conditions. This was achieved by carefully controlling the reactant ratios, catalyst concentrations, and reaction times (Scheme 8a).^83,84^ The reaction of borazine with 1-butene, *cis*-2-butene, and *trans*-2-butene yielded the same product 6, from this, the reaction exhibits high selectivity. Among them, terminal butene reacted the fastest, followed by the cis isomer, which reacted faster than the trans isomer (Scheme 8b). These compounds hold promise as processable precursors for boron nitride.

#### 3.1.2 Alkenylation reactions

**Scheme 9:**
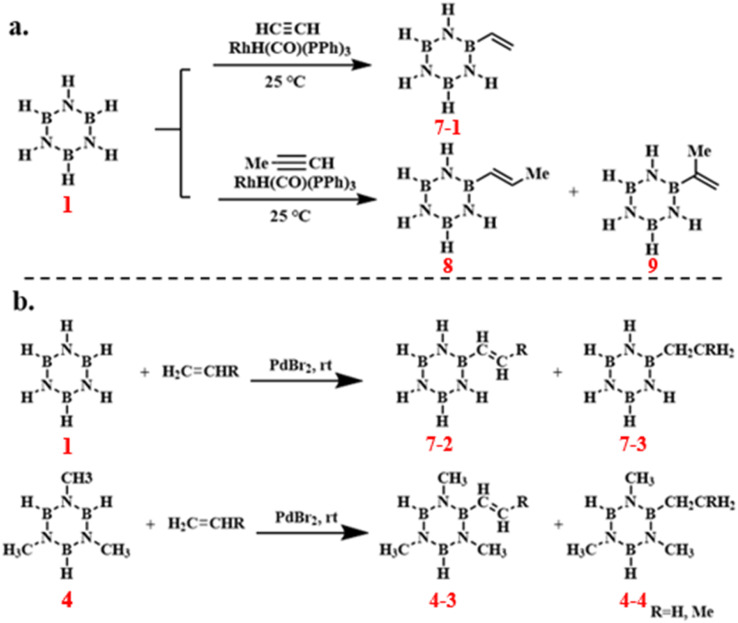
(a) RhH(CO)(PPh_3_)_3_-catalyzed reaction of borazine with alkynes; (b) PdBr_2_-catalyzed reaction of borazine and *N*-trimethyl borazine with alkenes.^85^

In 1987, A. T. Lynch and others pioneered a transition metal-catalyzed borane reaction, enabling the activation of B–H bonds and facilitating the alkyne addition reaction or dehydrogenative coupling of alkenes *via* boron-centered B–H bond alkenylation.^85^ Subsequent investigations into high-yield synthesis pathways for *B*-alkenyl borazines revealed that RhH(CO)(PPh_3_)_3_ catalysis enabled the addition of alkynes to borazine, yielding a diverse array of boron-substituted alkenyl borazines with yields reaching 70–85% (Scheme 9a). Additionally, PdBr_2_ catalysis promoted the dehydrogenative coupling of borazine and *N*-trimethyl borazine with alkenes, producing the corresponding *B*-vinyl and *B*-ethyl borazine products with yields up to 85% (Scheme 9b). It was discovered that *B*-alkenyl borazine can undergo thermally induced polymerization to form poly(alkenyl borazine) polymers, offering a novel approach and concept for the synthesis of boron nitride ceramic materials. By modulating the borazine content, it is possible to control the molecular weight and solubility of the polymer, thereby generating low-molecular-weight, soluble oligomers that hold promise for applications in film production or as coatings for ceramic materials.

**Scheme 10:**
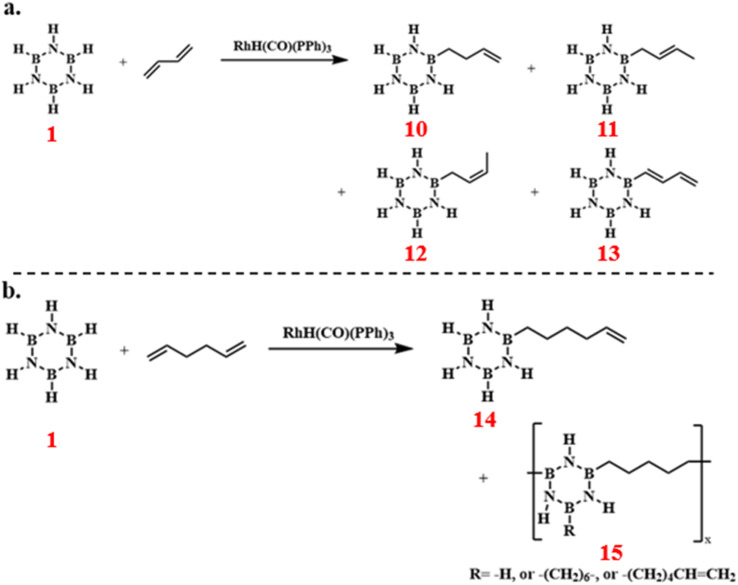
(a) RhH(CO)(PPh_3_)_3_-catalyzed reaction of borazine with 1,3-butadiene;^83^ (b) RhH(CO)(PPh_3_)_3_-catalyzed reaction of borazine with 1,5-hexadiene.^84^

Paul J. Fazen's team discovered that borazine reactions catalyzed by RhH(CO)(PPh_3_)_3_ with 1,3-butadiene and 1,5-hexadiene could produce *B*-substituted alkenyl borazine compounds.^83,84^ The reaction results indicated that under the given reaction conditions, 1,3-butadiene engaged in multiple reaction pathways, resulting in a complex mixture of products (Scheme 10a). In contrast, 1,5-hexadiene exhibited higher selectivity, predominantly yielding a single alkenyl product 14 (Scheme 10b). These compounds hold significant potential as processable precursors for the synthesis of boron nitride.

#### 3.1.3 Arylation reactions

**Scheme 11:**
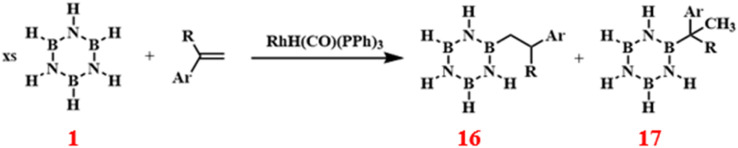
RhH(CO)(PPh_3_)_3_-catalyzed reaction of borazine with vinyl aromatics.^83,84^

Paul J. Fazen and others have developed a novel synthetic pathway for the preparation of high-performance boron nitride-based materials by synthesizing *B*-substituted borazine compounds with specific substitution patterns. This was achieved through RhH(CO)(PPh_3_)_3_-catalyzed reactions of 1 with vinyl aromatics, such as styrene, *p*-methylstyrene, and 4-allylphenyl ether, yielding products with 82% to 90% efficiency (Scheme 11).^83,84^

### 3.2 Hydroalkoxylation reactions

**Scheme 12:**
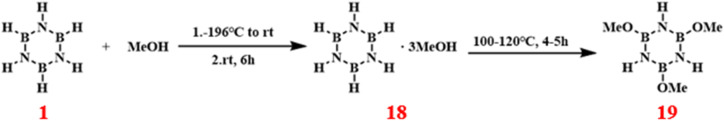
Synthesis route of *B*-trimethoxy borazine.^86^

In 1959, Haworth D. T. and others first studied in detail the thermolysis reaction of borazine–methanol adducts (Scheme 12).^86^ They proposed a reaction mechanism that involves the formation of intermediates such as 18. This finding challenged the earlier assumption that the reaction would produce monomers like CH_3_OBNH, thereby providing crucial data and theoretical support for a deeper understanding of the thermolysis reaction of borazine–methanol adducts.

### 3.3 Chlorination reactions

**Scheme 13:**
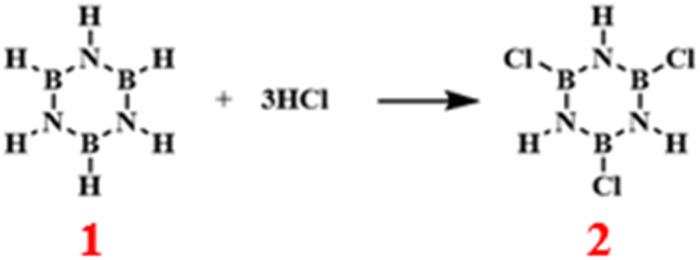
Reaction route of borazine with hydrogen chloride.^87^

Borazine, structurally akin to benzene, is highly reactive, and one of its most characteristic reactions is the addition reaction with hydrogen chloride. Upon reaction with hydrogen chloride, the nitrogen atom in borazine experiences electrophilic attack by H^+^, which cleaves the nitrogen-boron double bond. This is followed by the binding of Cl^−^ to the positively charged boron ion, resulting in the formation of 2 with a yield as high as 95% (Scheme 13).^87^

### 3.4 Bromination reactions

**Scheme 14:**
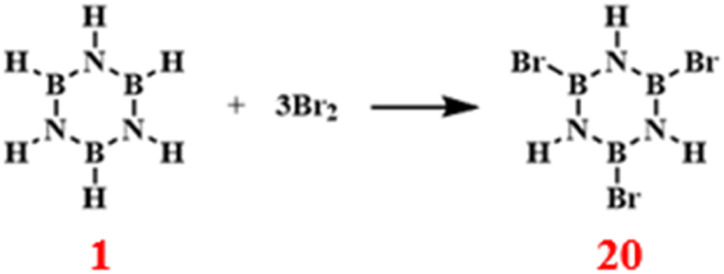
Reaction route of borazine with bromine.^87^

The reaction of borazine with bromine primarily occurs *via* electrophilic substitution reactions.^87^ Bromine, acting as an electrophile, preferentially attacks the para position of the nitrogen (N) ring or the *meta* position of the boron (B) ring in borazine (Scheme 14). This preference is due to the hydrogen atoms at the para position of the N ring being less influenced by the electronic inductive effect of the nitrogen atom, thus making them more susceptible to bromination. Conversely, the hydrogen atoms in the *meta* position of the B ring are less affected by the electron-withdrawing effect of the boron atom, rendering them more vulnerable to bromine attack. Reactions of borazine with other halogens, such as chlorine and iodine, exhibit similar mechanisms and generally produce adducts or substitution products.

### 3.5 Amination reactions

**Scheme 15:**
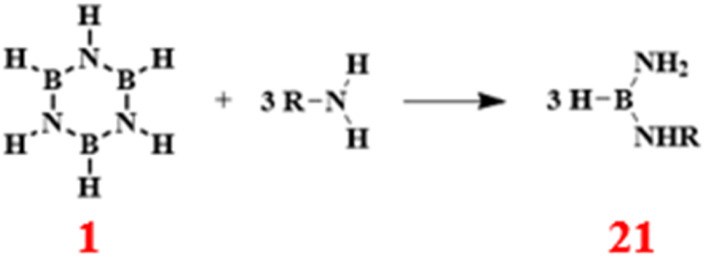
Reaction route of borazine with aromatic amines.^88^

In 1965, Alfred Kreutzberger and others initiated a systematic study of borazine ring-opening reactions with aromatic amines. For instance, the reaction with aniline produced *N*,*N*′-diphenyltriaminoborane with a yield of 69.5% (Scheme 15).^88^ This work unveiled the mechanism underlying boron–nitrogen bond formation, thereby providing fresh perspectives for the application of boron–nitrogen compounds in the realm of organic synthesis.

**Scheme 16:**
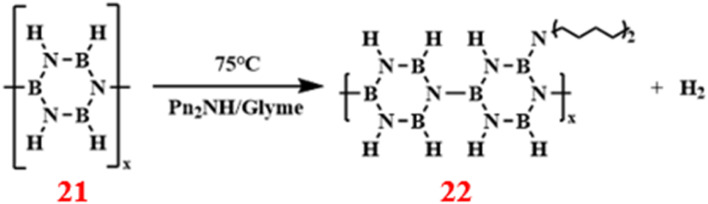
Synthesis route of dipentylamine-modified polyborazine.^89^

In 1996, Thomas Wideman and others introduced a dipentylamine-modified polyborazine (DPAPB) polymer precursor for the first time, with a yield of 91% (Scheme 16).^89^ By integrating dipentylamine into the borazine structure, they significantly reduce the polymer's melting temperature and postpone its crosslinking reaction, thus making it suitable for melt spinning. This advancement not only addressed the processing constraints of traditional polyborazine but also provided a new strategy for the production of high-performance ceramic fibers.

### 3.6 Reactions with silazanes

**Scheme 17:**
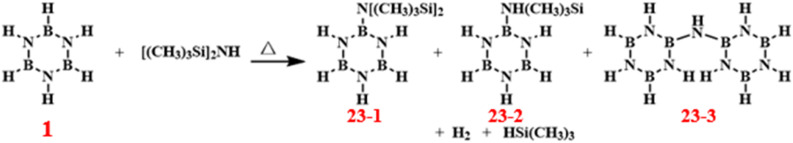
Synthesis route of borazine-modified hydrogenated polysilazane.^90^

In 1993, Kai Su and others developed a new type of borazine-modified hydrogenated polysilazane (HPZ) polymer (Scheme 17) as a precursor for the preparation of SiNCB (silicon nitride boron carbon) ceramic composite materials.^90^ It was found that the addition of borazine significantly improved the ceramic yield and thermal stability and also played a crucial role in inhibiting crystallization.

**Scheme 18:**
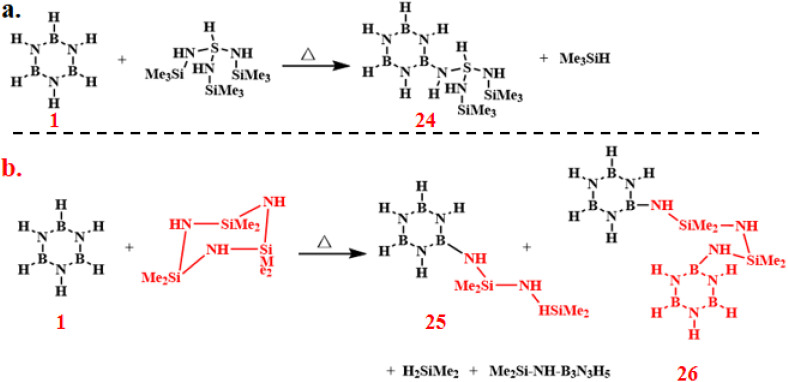
(a) Thermal condensation reaction of borazine with TTS; (b) thermal condensation reaction of borazine with HCT.^91^

In 1995, the same research group and others synthesized a series of borazine/silazane copolymers through the thermal condensation reaction of borazine with two silazanes (tris(trimethylsilyl)silane (TTS) and 1,1,3,3,5,5-hexamethyltrisiloxane (HCT)) as single-source polymer precursors for the preparation of SiNCB ceramic composite materials (Scheme 18a and b).^91^ The results showed that by adjusting the composition of the copolymers, the composition and properties of the resulting ceramic materials could be controlled. Although the ceramics derived from the two copolymers exhibited different compositions and crystallization characteristics at high temperatures, they all demonstrated excellent thermal stability and amorphous features. These modified polymers, due to their ease of synthesis and processability, as well as the superior properties of the derived ceramics, hold great potential as precursors for high-temperature ceramic materials.

## 4. Synthesis of functionalized borazine derivatives

**Scheme 19:**
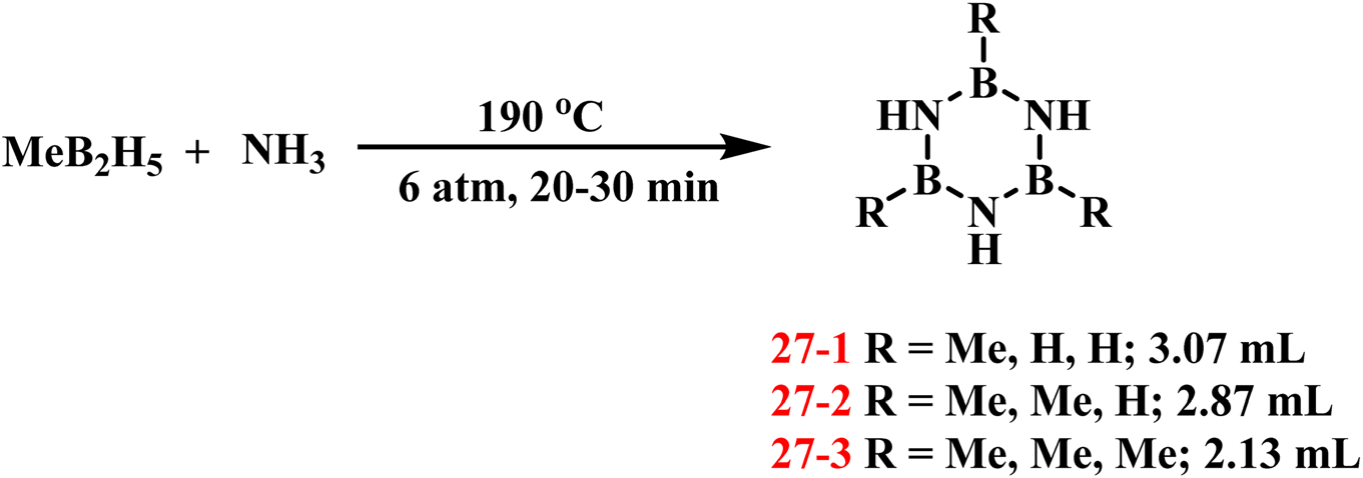
Synthesis of substituted borazine derivatives by the one-step reaction of MeB_2_H_5_ and NH_3_.^92^

In 1936, Schlesinger, Burg, and coworkers achieved the first synthesis of alkyl-substituted borazine derivatives (27) through the reaction of MeB_2_H_5_ with ammonia NH_3_ at 0 °C under 6 atm pressure for 20–30 min (Scheme 19).^92^ The crude products were isolated *via* condensation distillation, yielding borazine derivatives with varying degrees of methyl substitution in different proportions. Subsequent attempts to react di-, tri-, and tetramethylboranes with NH_3_ under analogous conditions were unsuccessful. The formation of multiple side products and inherent challenges in isolating the target compounds precluded the preparation of alkyl-substituted borazine derivatives from these higher methylated boranes.

**Scheme 20:**
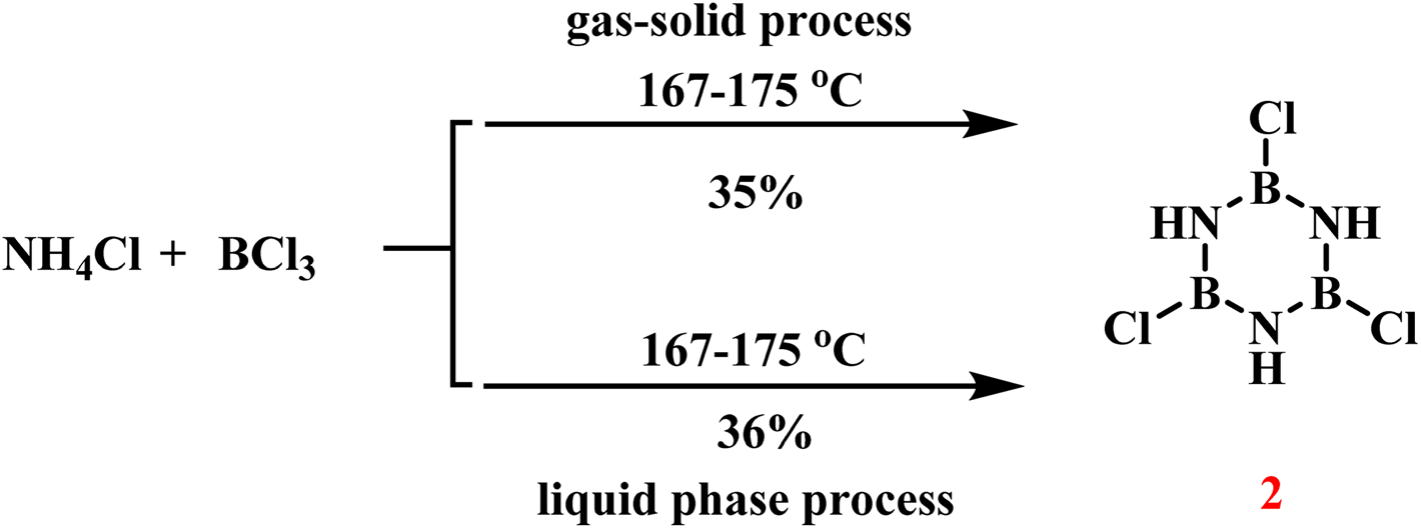
Gas–solid and liquid phase methods for the synthesis of 2.^93^

In 1955, Brown and coworkers developed two distinct synthetic routes to 2 (Scheme 20).^93^ The gas–solid phase method involved reacting anhydrous ammonium chloride (NH_4_Cl) powder with boron trichloride (BCl_3_) vapor at 167–175 °C under nitrogen atmosphere, yielding limited quantities of colorless 2 crystals (35% yield). Additionally, they mixed NH_4_Cl with BCl_3_ in a solvent and heated the mixture under reflux, achieving a 36% yield of 2.

**Scheme 21:**
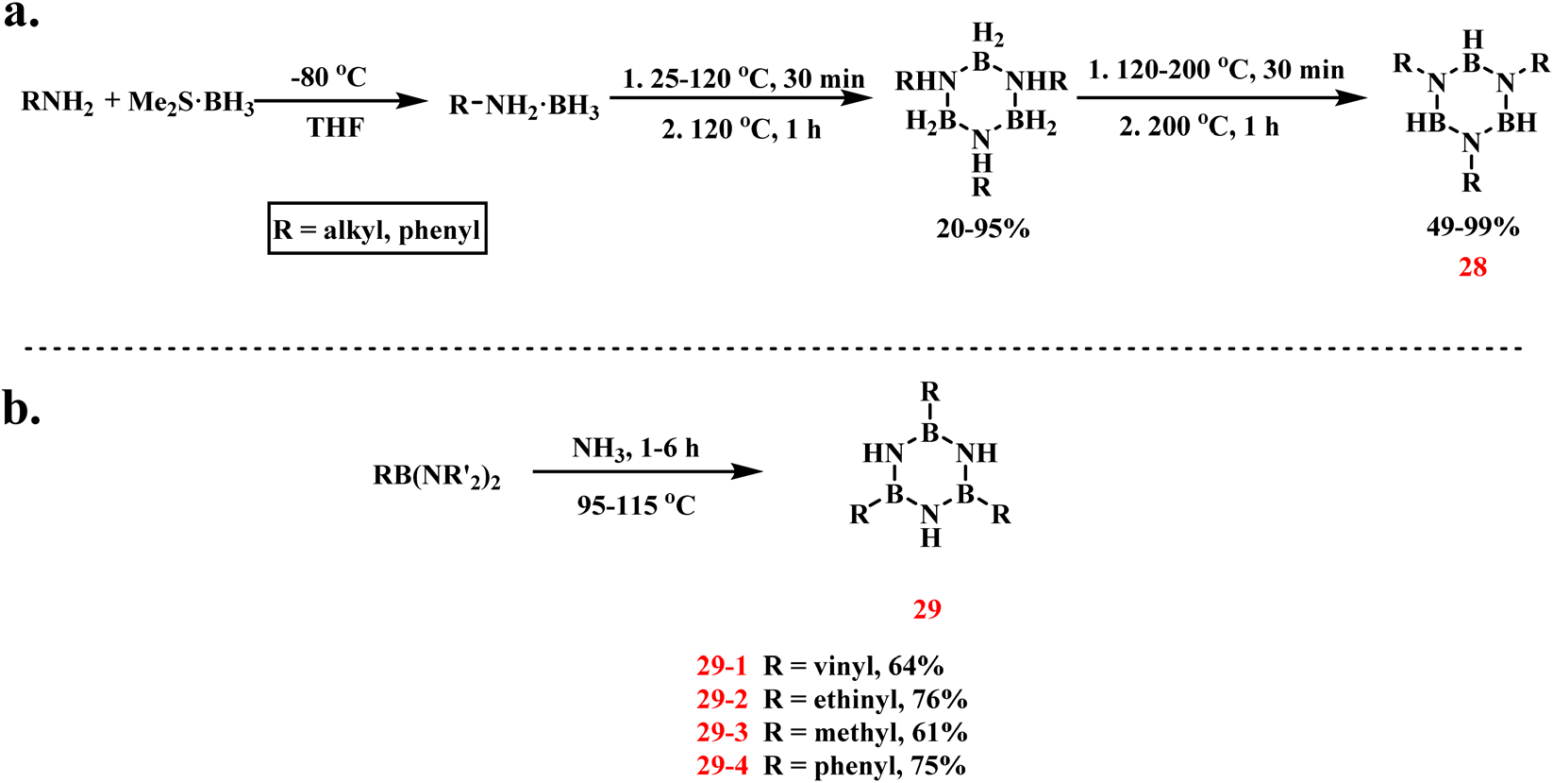
(a) Synthesis of *N*-trisubstituted borazine by reaction of primary amine with Me_2_S·BH_3_;^94^ (b) synthesis of *B*-trisubstituted borazine by heating the ammonia elimination of bis(diisopropylamino)organoboranes.^96^

In 2000, Vaultier and Framery systematically refined the pyrolysis protocol for amine–borane complexes (RNH_2_·BH_3_), building upon Brown's foundational work to achieve efficient synthesis of *N*-substituted borazines (Scheme 21a).^94,95^ The synthetic sequence commenced with the formation of alkyl/aryl amine–borane complexes through the reaction of Me_2_S·BH_3_ with amines in THF at −80 °C, delivering products in 90–99% yield. Final cyclization to 28 was accomplished by heating the intermediates at 200 °C for 1 h. Critical advancements over prior methodologies included the strategic replacement of thermally labile THF·BH_3_ with Me_2_S·BH_3_ as a stabilized boron source. Furthermore, Fritz's ammonia elimination protocol was enhanced through the addition of 1 mol% phenothiazine, which elevated the yield of diethylamine ethylborane-derived products (29-1) to 64% under equivalent conditions.^96^ This approach was successfully generalized to bis(diisopropylamino)organoboranes, enabling the synthesis of *B*-alkyl, *B*-vinyl, and *B*-alkynyl borazine derivatives with 64–76% isolated yields (Scheme 21b).

**Scheme 22:**
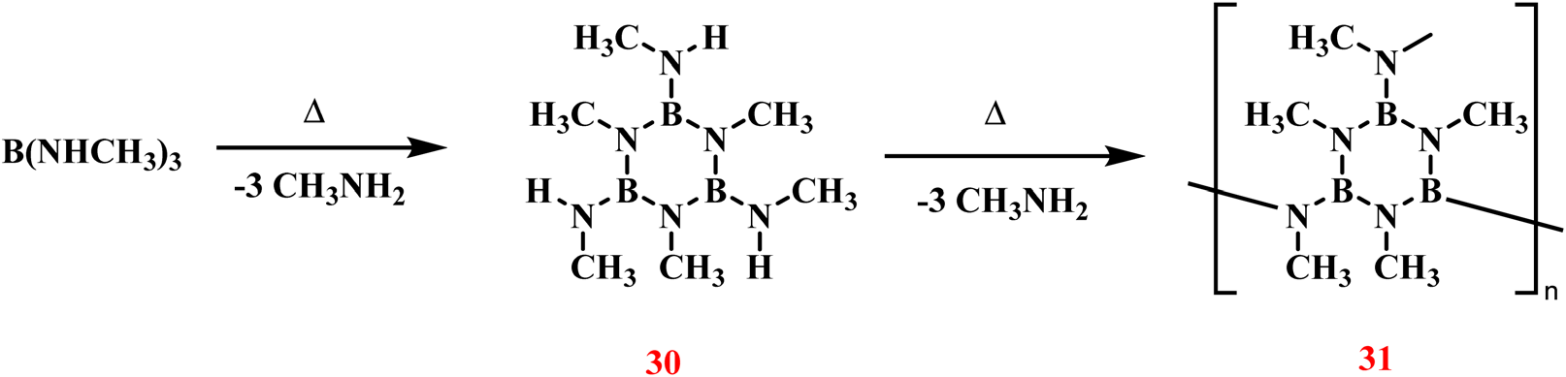
Thermal elimination of MeNH_2_ from B(NHCH_3_)_3_ to form polymer.^97^

Cornu and coworkers conducted a systematic investigation into the structural effects of substituent groups (R) on the ceramic yield of boron nitride fibers derived from B(NHR)_3_ precursors (Scheme 22).^97^ The synthetic pathway involved high-temperature pyrolysis of these precursors, with concomitant elimination of RNH_2_ molecules to generate cross-linked polymeric intermediates. Among the tested substituents, methyl-functionalized derivatives (R=CH_3_) exhibited optimal performance, achieving the maximum ceramic yield under identical pyrolysis conditions.

**Scheme 23:**
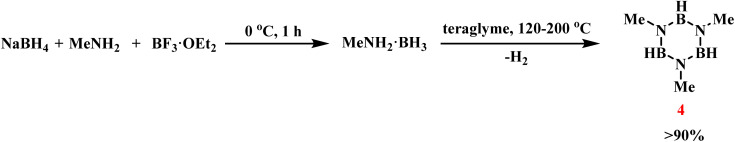
Synthesis of 4 using NaBH_4_, MeNH_2_, and BF_3_·OEt_2_.^98^

In 2006, Yamamoto and coworkers reported a high-yielding synthesis of 4 from NaBH_4_, methylamine MeNH_2_, and BF_3_·OEt_2_ (Scheme 23).^98^ The protocol involved sequential addition of BF_3_·OEt_2_ to a THF solution containing NaBH_4_ and MeNH_2_ at 0 °C, followed by 1 hour reaction. Subsequent removal of volatiles (excess MeNH_2_ and THF) by evaporation yielded a crude intermediate, which was redissolved in tetraglyme for thermal treatment. Gradual heating to 200 °C induced dehydrogenative coupling, affording 4 in 94% isolated yield. While the method risks potential contamination by fluoroborane byproducts, a stoichiometric excess of NaBH_4_ effectively suppressed their formation. The dual role of NaBH_4_ as both a boron source and a side-reaction inhibitor, coupled with its cost-effectiveness, renders this approach particularly advantageous for scalable synthesis.

**Scheme 24:**
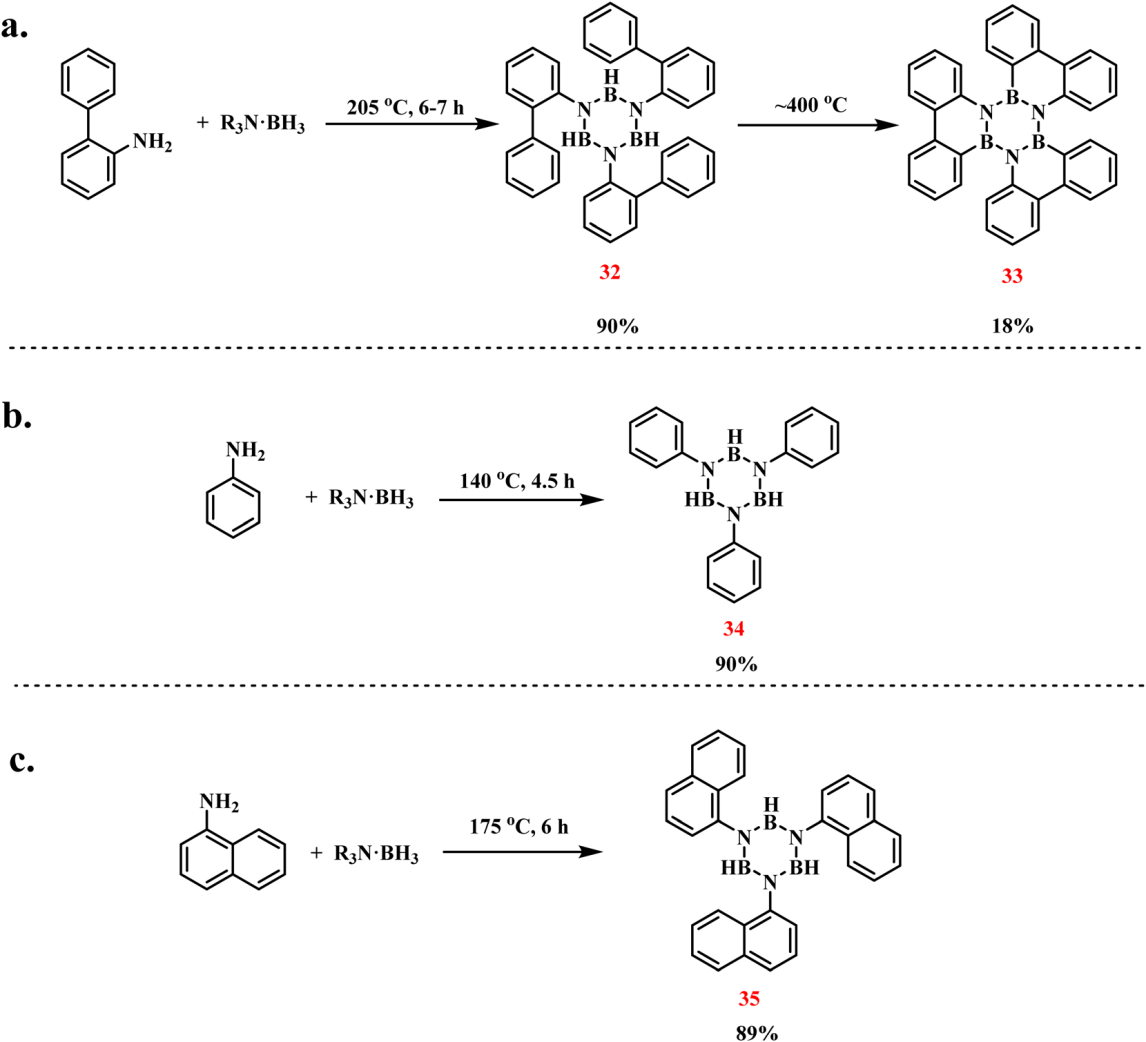
Dehydrogenative coupling of primary amines with triethyl aminoborane to prepare substituted borazines: (a) 2-aminobiphenyl;^99^ (b) phenylamine;^100^ (c) α-naphthylamine.^100^

Köster and coworkers developed a thermal dehydrogenation strategy for synthesizing *N*-triarylated borazines through amine–borane coupling (Scheme 24).^99^ Treatment of 2-aminobiphenyl with triethyl aminoborane at 205 °C under argon for 6–7 h afforded *N*-tri(biphenyl)borazine (32) in 90% yield, accompanied by H_2_ and triethylamine liberation. Subsequent thermal treatment of this intermediate at 400–410 °C for 6.5 h induced boron-bound hydrogen elimination and biphenyl coupling, yielding triphenylborazine (33) as yellow needle-like crystals (18% yield). Single-crystal X-ray analysis revealed a propeller-like spatial arrangement of the biphenylylene substituents. In parallel experiments, analogous dehydrogenative coupling of phenylamine and α-naphthylamine with triethyl aminoborane under identical conditions produced *N*-triphenylborazine (34, 90%) and *N*-trinaphthylborazine (35, 89%), respectively (Scheme 24b and c).^100^ These results systematically demonstrate the generalizability of thermally driven dehydrogenative coupling between primary aryl amines and amine–boranes for constructing trisubstituted borazine architectures.

**Scheme 25:**
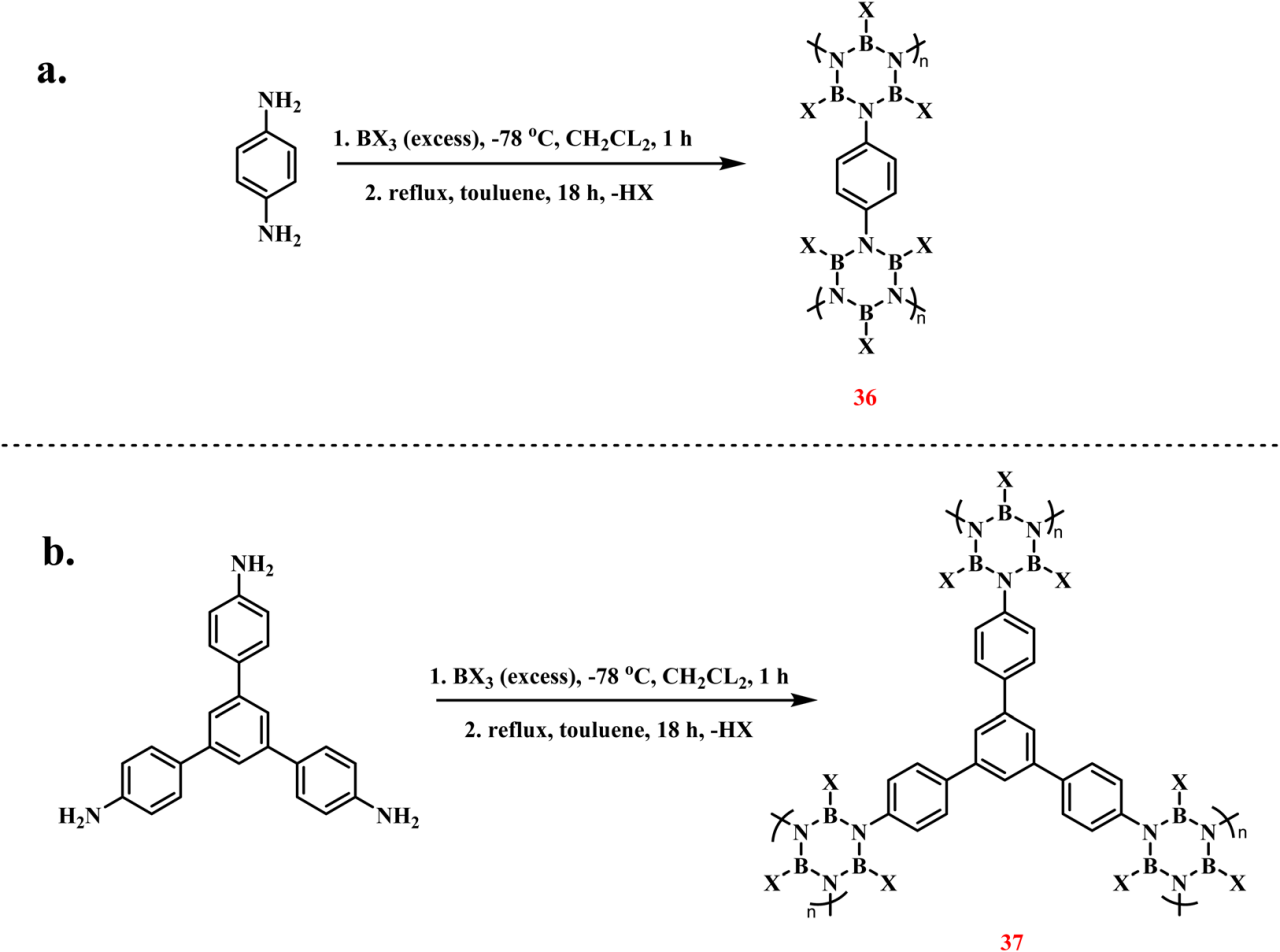
Synthesis of functionalized borazine polymers *via* pyrolysis of arylamines with boron trihalides: (a) 1,4-phenylenediamine; (b) 1,3,5-tris(4-aminophenyl)benzene.^101^

H. M. El-Kaderi *et al.* synthesized borazine-core porous polymers *via* condensation of arylamines with boron trihalides (Scheme 25).^101^ The synthesis involved reacting 1,4-phenylenediamine with excess boron trihalide (BCl_3_ or BBr_3_) at −78 °C for 1 h, followed by HX (X = Cl/Br) elimination *via* 18 h toluene reflux, ultimately yielding white polymeric powders in high yield. Structural analysis confirmed the porous architecture of the polymers, which demonstrated notable gas adsorption capabilities. Comparative studies revealed that the Cl-system exhibited superior porosity and hydrogen adsorption performance compared to the Br-analogue, attributed to steric hindrance caused by the bulkier bromine atoms. When 1,3,5-tris(4-aminophenyl) benzene was employed as the monomer, analogous borazine-embedded porous polymers were successfully obtained.

**Scheme 26:**
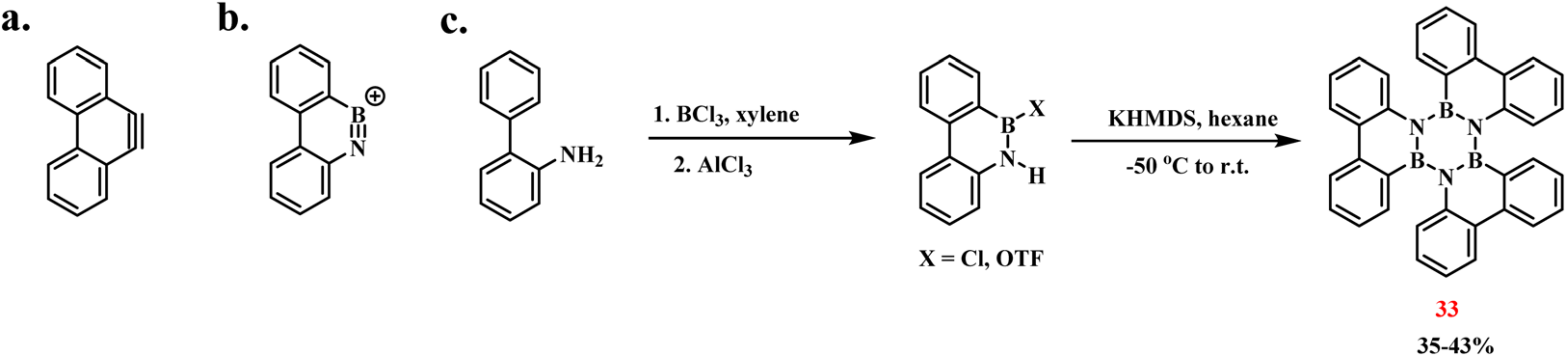
(a) 9,10-phenanthryne; (b) 9,10-azaboraphenanthryne; (c) synthesis of triphenylborazine by Bettinger *via* dehydrohalogenation.^102^

Since 33 serves as the trimer of 9,10-azaboraphenanthryne—a BN isostere of 9,10-phenanthryne—Bettinger developed an optimized synthesis *via* dehydrohalogenation to achieve enhanced yields (Scheme 26a and b).^102^ Through the action of a base, HCl can be eliminated from 9-chloro-9-bora-10-azaphenanthrene, or HOTF can be eliminated from the corresponding triflate, leading to the BN–aryne cyclic trimer-triphenylborazine. The yield of 33 obtained through the dehydrohalogenation was 35%, while by replacing the chloride with the HTOF of 9-chloro-9-bora-10-azaphenanthrene, the yield was improved to 43% (Scheme 26c).

**Scheme 27:**
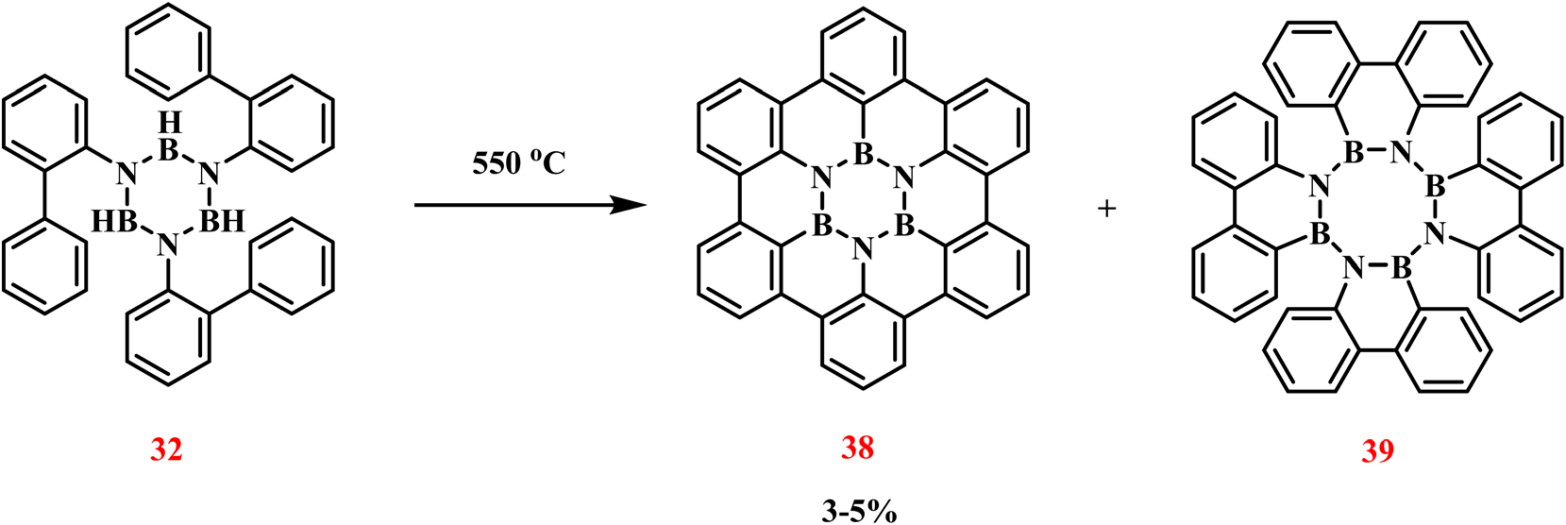
Synthesis of 38.^103^

Building upon Köster's methodology, Bettinger achieved the synthesis of BN–hexa-*peri*-hexabenzocoronene (38) through controlled thermal treatment of 32 (Scheme 27).^103^ Stepwise heating to 550 °C induced dehydrogenative coupling, forming 38 with a central B_3_N_3_ cyclic core. Initial melting occurred at 350 °C, producing a colorless liquid phase. Sustained heating at 550 °C for 2 h induced solidification, with subsequent 4 h annealing and gradual cooling yielding a black matrix containing surface-deposited colorless crystals. Single-crystal X-ray diffraction unambiguously identified these crystals as 38, though tetraazatetraborocine derivatives (39) constituted the primary products (>95%). Three-stage Soxhlet extraction recovered 3–5% 38, demonstrating limited process efficiency. Despite low yields, the synthesized 38 exhibited structural features conducive to boron nitride-doped graphene fabrication, highlighting its potential for organic electronic applications.^104^

**Scheme 28:**
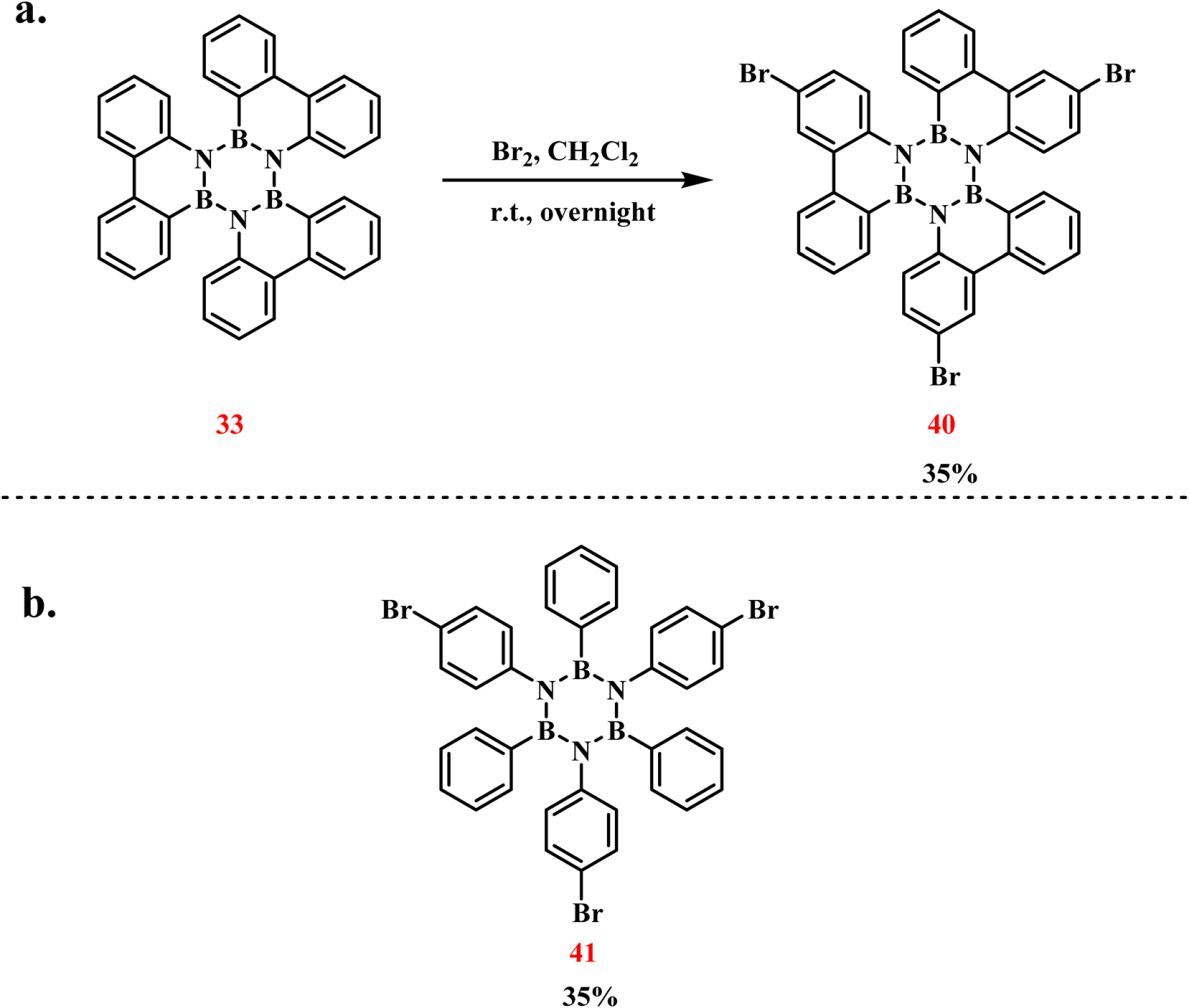
(a) Bromination reaction of triphenylborazine; (b) bromination reaction of hexaphenylborazine.^105^

In 2013, Bettinger's group achieved the first synthesis of tribrominated triphenylborazine derivatives (40) *via* electrophilic aromatic bromination of arylborazines with elemental bromine (Scheme 28).^105^ The reaction involved dissolving 33 in CH_2_Cl_2_ under an argon atmosphere, followed by 12 hours stirring at room temperature. Steric hindrance imparted by the three phenyl groups surrounding the B_3_N_3_ core directed Br_2_ to undergo regioselective functionalization, yielding the monobrominated triphenylborazine derivative (41) in 35% isolated yield.

**Scheme 29:**
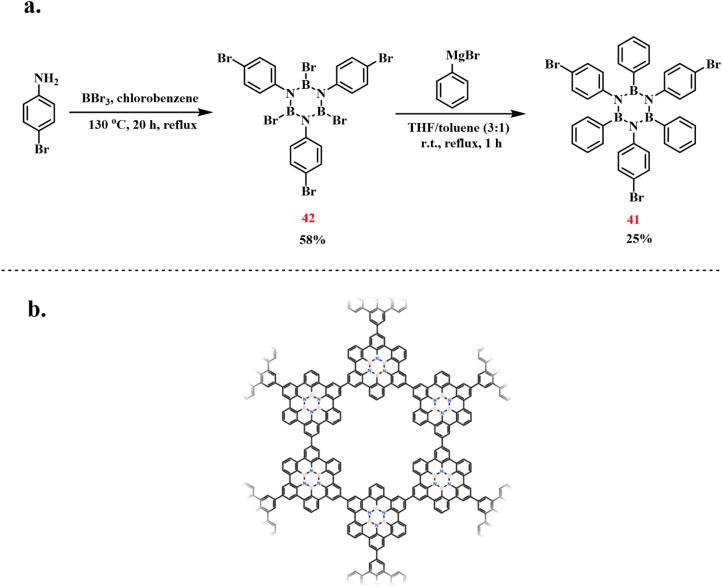
(a) Synthesis of 41 from 42 and BBr_3_; (b) aryl coupling of 41 on the Ag (111) surface to form a 2D network.^106^

In 2015, the same group successfully group reported an alternative synthesis of 41*via* sequential functionalization (Scheme 29a).^106^ The protocol initiated with dehydrogenative coupling of 4-bromoaniline and BBr_3_ under thermal conditions, yielding *B*-tribromo-*N*-tris(4-bromophenyl)borazine (42). Subsequent treatment of this intermediate with phenylmagnesium bromide (PhMgBr) in a mixed THF/toluene solvent system selectively replaced N-bound bromine atoms with phenyl groups, affording 41 in 25% isolated yield. Additionally, by utilizing the three bromine substituents, the monomer was placed on an Ag (111) surface. An aryl coupling reaction then formed a two-dimensional polymer network, which holds potential for applications in electronics and optics (Scheme 25b).

**Scheme 30:**
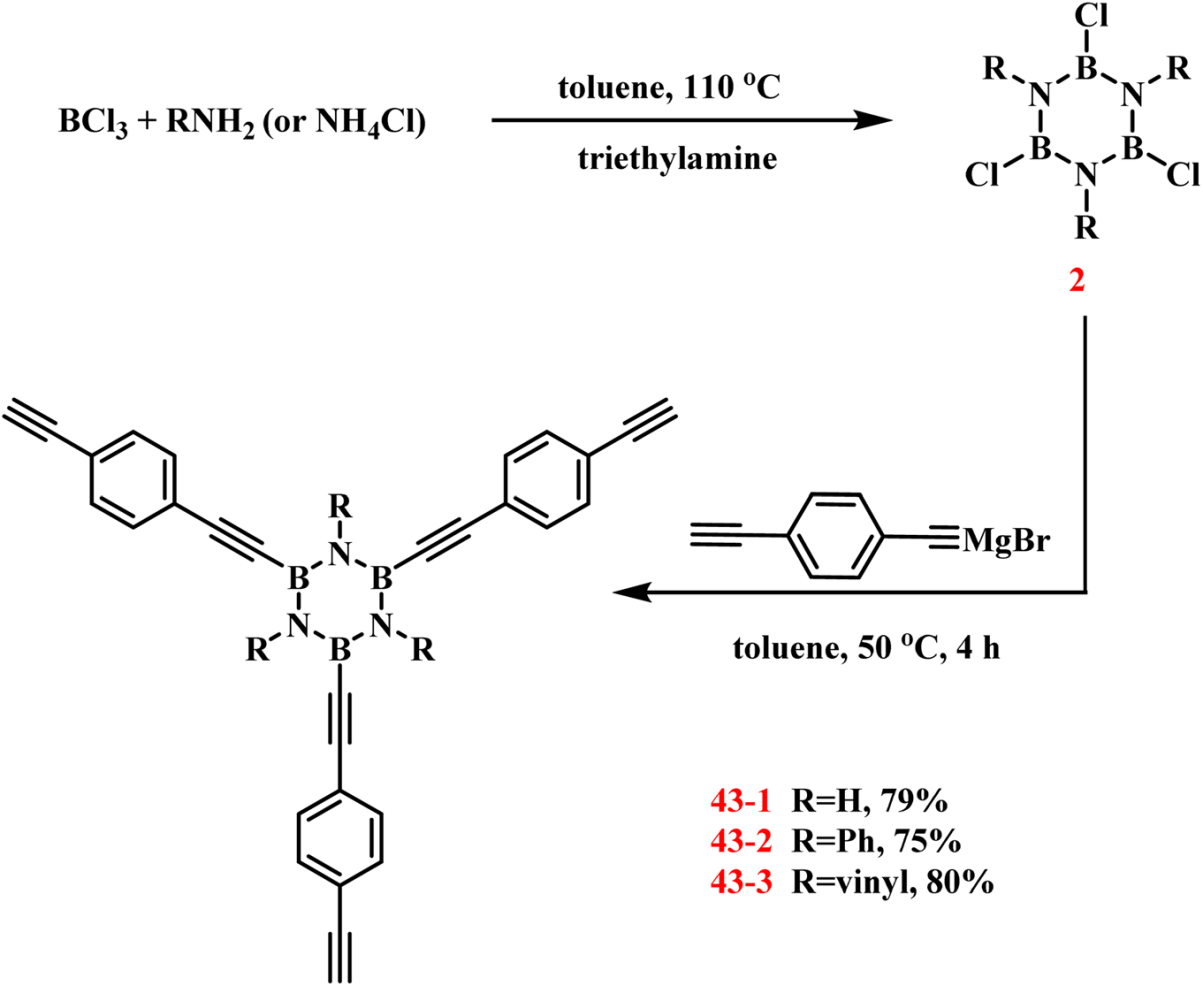
Synthesis of borazine derivatives by Grignard reagent substitution of 2.^107^

Qi developed a synthetic route to borazine-containing arylacetylene resins (43) through sequential functionalization (Scheme 30).^107^ The process commenced with the preparation of 2*via* reaction of BCl_3_ with primary amines or NH_4_Cl in toluene at 110 °C, establishing the essential boron–nitrogen ring framework. A THF solution of ethynylphenylethynyl magnesium bromide was then treated with the 2/toluene mixture under nitrogen at 0 °C by dropwise addition. Gradual heating to 50 °C with 4 hours stirring yielded 43-1 as a brown viscous liquid (79% yield). An analogous procedure afforded 43-2 in 75% yield. The authors cross-linked these monomers to obtain the corresponding resins, which exhibit good properties and have potential applications in microelectronic packaging, radio and microwave frequency substrates.

**Scheme 31:**
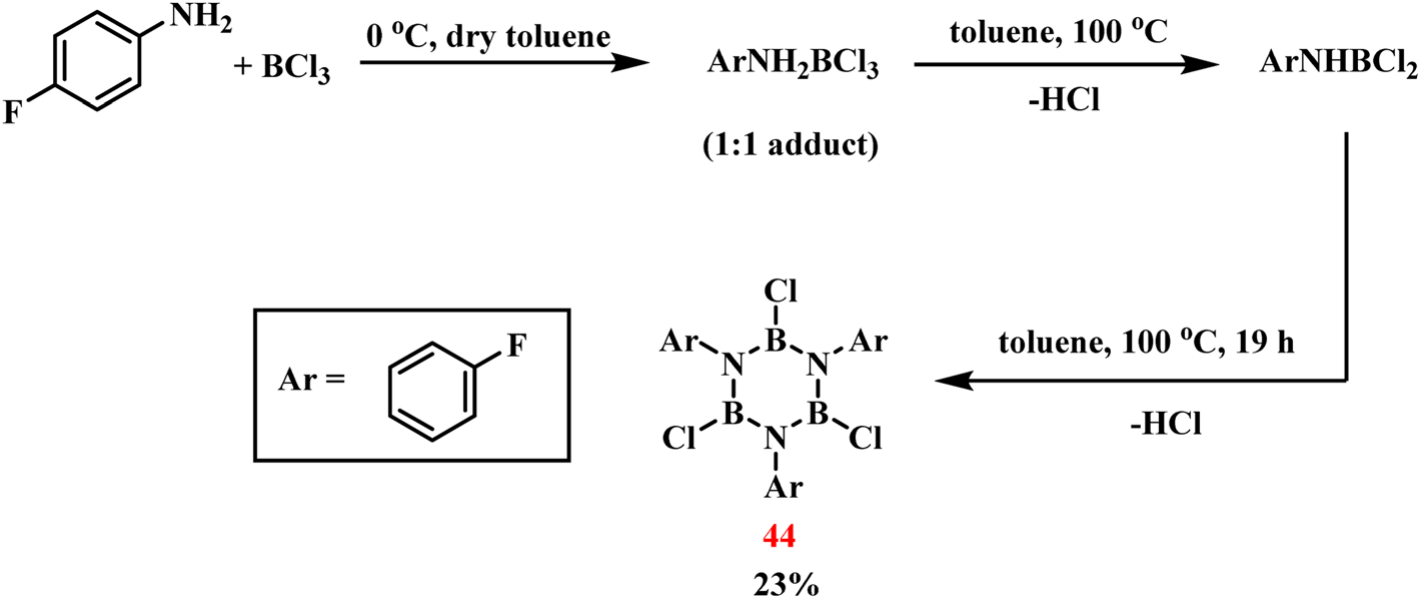
Synthesis of 44*via* pyrolysis of *p*-fluoroaniline with BCl_3_.^108^

In 2018, Bettinger's group established a stepwise synthesis of *B*-trichloro-*N*-tri(*p*-fluorophenyl)borazine (44) through controlled dehydrochlorination of *p*-fluoroaniline (ArNH_2_) and BCl_3_ (Scheme 31).^106^ The initial reaction of ArNH_2_ with BCl_3_ in toluene at 0 °C produced a 1 : 1 Lewis acid–base adduct (ArNH_2_·BCl_3_), as confirmed by single-crystal X-ray analysis (B–N bond: 1.61 Å; B–Cl bonds: 1.84 Å). Sequential thermal treatment first at 100 °C induced HCl elimination, generating the transient intermediate ArNHBCl_2_, which was characterized by NMR spectroscopy despite its inherent instability. A subsequent heating cycle promoted further HCl elimination, ultimately yielding the target compound in 23% yield. Kinetic studies identified the reaction between ArNHBCl_2_ and free ArNH_2_ as the rate-determining step of the process.

**Scheme 32:**
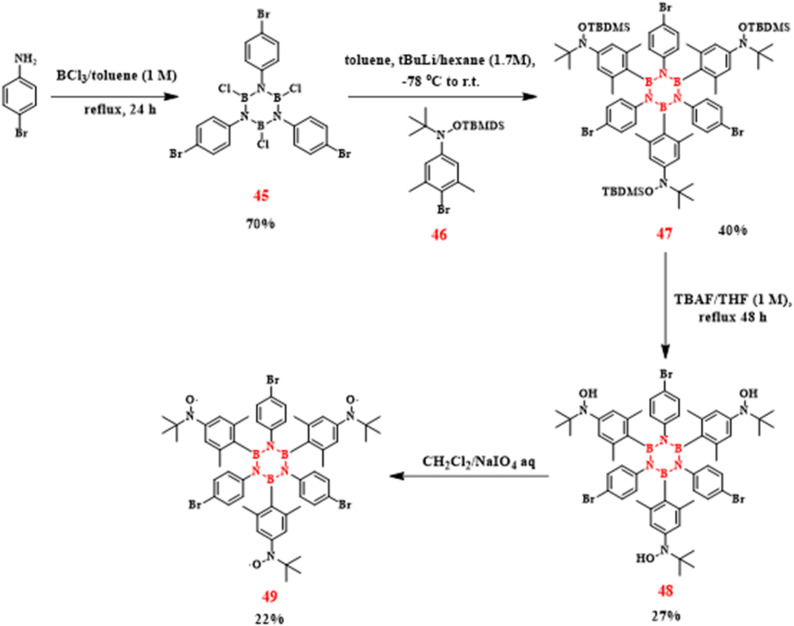
Synthesis route of 49.^109^

In 2024, Dominique Luneau's team achieved the first synthesis of open-shell borazine derivatives through trifunctional nitroxide radical ligation to boron centers (Scheme 32).^109^ The synthesis began with a 24 hours reflux of 4-bromoaniline and BCl_3_, yielding the precursor *B*-trichloro-*N*-tris(4-bromophenyl)borazine (45). Chlorine substituents on the boron were then replaced by reaction with silyl-protected *N*-(4-bromo-3,5-dimethyl)-*N-tert*-butylhydroxylamine hydrochloride (46). Tetrabutylammonium fluoride (TBAF) treatment under reflux caused Si–O bonds to be selectively broken, releasing hydroxyl groups. Nitroxide radicals were then stabilized by NaIO_4_-mediated oxidation. This sequence furnished the target compound *N*-tris(4-bromophenyl)-*B*-tris(2,6-dimethyl-4-(*N-tert*-butyl-*N*-oxyamino)phenyl)borazine (49) with 22% overall yield. By integrating radical functionalities into the inorganic aromatic framework, this work establishes a novel platform for spin-state engineering, demonstrating potential for advanced magnetic material development.

**Scheme 33:**
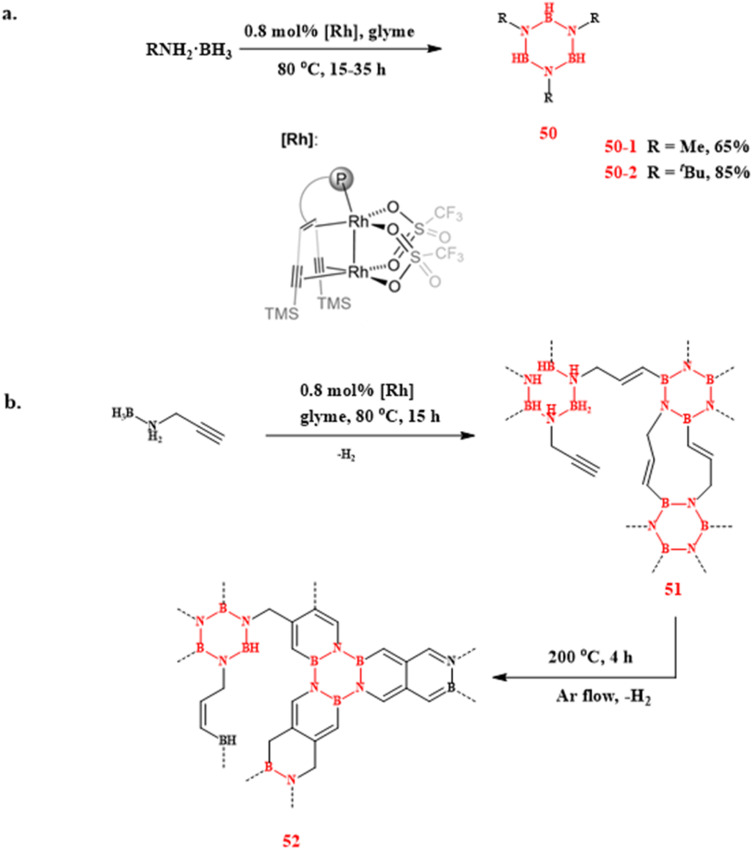
(a) Dehydrogenation of ammonia borane with rhodium olefin complexes (P = PPh_2_). (b) Rhodium–olefin complex-catalyzed BCN synthesis from *N*-propargylamine–borane conversion.^110^

In the same year, the research team led by Hansjörg Grützmacher reported a highly efficient synthetic protocol for borazine derivatives catalyzed by a binuclear olefin–rhodium complex (Scheme 33).^110^ The optimized procedure involved adding 0.8 mol% [Rh] catalyst to a glyme solution containing primary amines, followed by heating at 80 °C for 15–35 h to obtain target borazines in high yields. Notably, this catalytic system exhibited dual functionality in the *N*-propargylamine–borane conversion: in addition to facilitating dehydrogenative coupling, it also promoted the formation of BCN-based polymeric products with controlled cross-linking density. These boron–carbon–nitrogen (BCN) network materials show promising potential as advanced coatings for metallic substrates.

## 5. Borazine-based functionalized materials

**Fig. 3:**
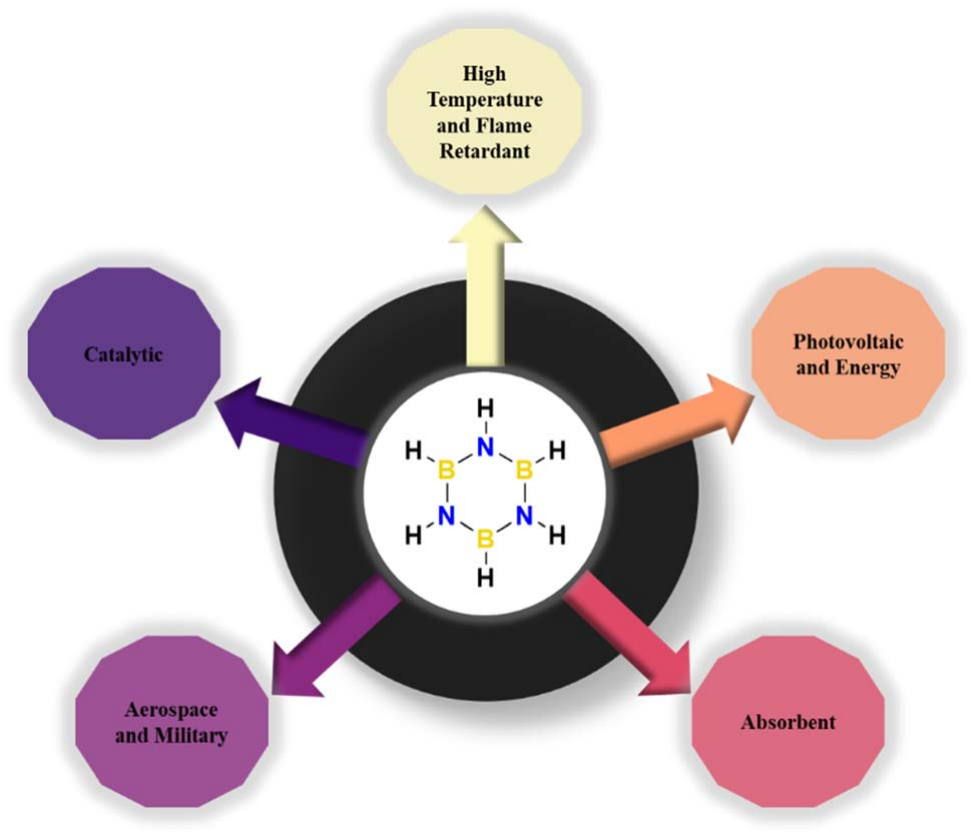
Various applications of borazine-based polymeric macromolecules in different fields.

The previous section describes the methodological techniques for the synthesis of borazine-based monomers and polymers, as well as the structural features of borazine. Next, the performance of borazine-based polymers in terms of thermal, mechanical, optical, catalytic, and adsorptive properties (Fig. 3) and their potential applications in aerospace, electronics, energy and the environment are highlighted.

### 5.1 Heat-resistant and flame-retardant materials

Borazine based polymers have excellent flame-retardant properties due to their high thermal stability and release of non-flammable gases during decomposition, and the formation of protective ceramic-like residues makes these polymers highly effective in preventing or mitigating the spread of fire. Polyborazine (PBN) can be converted at high temperatures to boron nitride (BN) ceramics for the production of high-temperature-resistant coatings or fibers. In addition, the flame-retardant properties of cyclic boron nitrides give them significant potential for use in fire-resistant materials.

**Fig. 4:**
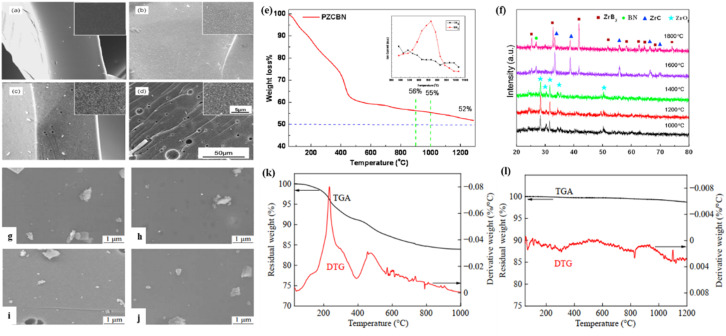
SEM images of Zr–C–B–N ceramics treated at different temperatures: (a)–(d), 1200, 1400, 1600 and 1800 °C. (e) MS curves for PZCBN precursor in Ar. (f) XRD curves of the as-pyrolyzed Zr–C–B–N ceramics at different temperatures. Reproduced from ref. 113, with permission of *Ceram. Int.*, copyright 2018. SEM images of PBSZ after sintering at (g) 1000 °C, (h) 1200 °C, (i) 1400 °C, and (j) 1500 °C. (k) TGA and DTG curves of cured PBSZ at 1000 °C. (l) TGA and DTG curves of SiBCN ceramics at 1200 °C in an air atmosphere. Reproduced from ref. 51, with permission of *Chem. Rev.*, copyright 2018.

Using borazine compounds, notably *B*-triethynyl-*N*-trimethylborazine and unsubstituted borazine, Jeon *et al.* first synthesized boron carbide ceramic prepolymers in 2004 using a catalyst-free hydroboration technique.^111^ A major breakthrough in the synthesis of ceramics generated from precursors was made when these hydroboration copolymers were successfully pyrolyzed to create homogenous amorphous boron carbide ceramics. Numerous studies on precursor design and processing optimization have been sparked by this discovery. A different synthesis route for boron nitride ceramic precursors was recently demonstrated by Zou's research group. They created borazine precursors using lithium borohydride and ammonium sulfate as starting materials, which, when pyrolyzed at 1400 °C, produced an impressively high ceramic yield of 89.5%. This is especially useful for creating boron nitride matrix composites.^112^ The reduction of structural flaws during the polymer-to-ceramic conversion process depends on this high ceramic retention. Ge *et al.*, who examined the synergistic effects of incorporating trichlorocycloborazine (TCB) into polycarbosilane (PCS) systems, reported additional advancements in precursor modification. According to their research, TCB additions greatly increased the high-temperature stability of PCS-derived materials while also increasing their ceramic production. New ideas for creating sophisticated ceramic systems with specialized thermomechanical properties are offered by this composite technique. Wang *et al.* recently produced a novel zirconium-containing preceramic polymer, polyzirconocenyborazane (PZCBN), by controlling the polymerization of bis(cyclopentadienyl)zirconium divinyl with borazine.^113^ This novel synthesis technique involves the simultaneous inclusion of Zr, B, C, and N components at the molecular level, resulting in a multi-component precursor for advanced ceramic systems. Microstructural evolution investigations (Fig. 4a–d) show remarkable sintering behavior between 1200 and 1800 °C. The resulting ceramics have unusually delayed grain coarsening kinetics and maintain high densification rates. These morphological traits point to inherent suppression of crystal formation pathways, which is a crucial benefit for structural applications involving high temperatures. A 52% ceramic yield at 1200 °C is revealed by thermogravimetric-mass spectrometric (TG-MS) analysis (Fig. 4e), which also shows staged pyrolysis processes suggestive of controlled thermal degradation. Temperature-dependent XRD phase stability studies (Fig. 4f) show no discernible phase changes up to 1800 °C, and oxidative thermogravimetric analysis shows better air stability than traditional Zr-free systems. High elemental homogeneity, inhibited grain growth, and remarkable thermochemical stability work in concert to make the Zr–C–B–N multi-phase ceramics made from PZCBN attractive options for next-generation ultrahigh-temperature ceramic composites that must endure extended use in harsh conditions.

Qi's research group established an efficient route for synthesizing polyborosilazane (PBSZ) precursors through nucleophilic substitution between trichloroborazine and ethynylmagnesium chloride, followed by high-temperature cracking.^51^ Defect-minimized ceramic surfaces at four pyrolysis temperatures (1000–1500 °C) are revealed by microstructural analysis (Fig. 4g–j), which also shows impressive densification with submicron-scale pore dispersion. Effective crosslinking during thermosetting and the ensuing ceramization processes is confirmed by this morphological change. Thermoanalytical studies show remarkable efficiency in converting precursors to ceramic. In comparison to traditional polymer-derived ceramic systems, thermogravimetric analysis (TGA) with derivative thermogravimetry (DTG) monitoring (Fig. 4k) records an 84.3% ceramic yield at 1000 °C in a nitrogen atmosphere. Fig. 4l shows that SiBCN ceramics exhibit near-perfect mass retention in air (99.0% at 1000 °C, 98.6% at 1200 °C), creating previously unheard-of oxidation resistance. Under severe thermal-oxidative circumstances, stabilized surface passivation mechanisms are suggested by the negligible 0.4% mass loss differential between 1000 and 1200 °C. PBSZ-derived ceramics are excellent options for thermal protection systems and other applications requiring precisely shaped components with nanoscale defect control because of their performance features, which include high ceramic yield and ultrastable high-temperature behavior. Long and colleagues recently provided an overview of the state of research on the organic precursor approach for manufacturing boron nitride ceramics.^114^ An outlook on the development of boron nitride precursors is provided, along with a description of the properties and uses of the ensuing boron nitride materials and the methods for creating them from borazine, boron trichloride, trichloroborazine, and other pathways. In summary, this advancement in flame retardant technology positions borazine polymers as enablers of safer, multi-purpose materials. The ceramic conversion capacity also allows for dual functionality: temporary polymer flame suppression followed by permanent BN ceramic protection, making PBN excellent for composites requiring both processability and severe fire resistance.

### 5.2 Photovoltaic and energy materials

**Fig. 5:**
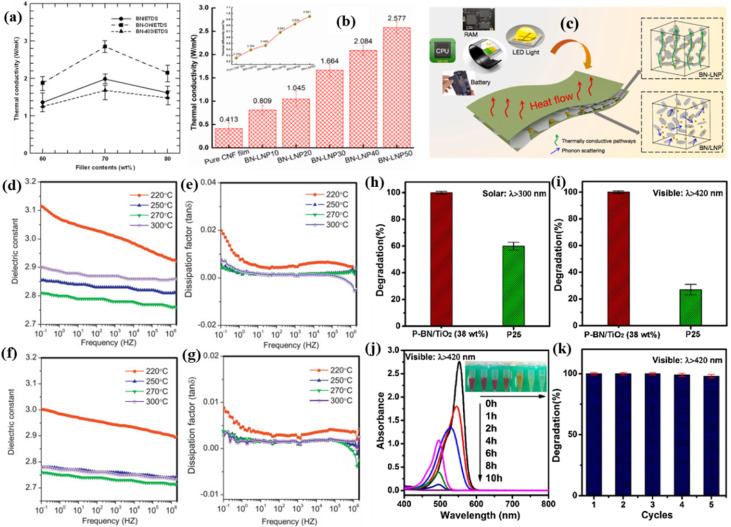
(a) Effect of surface treatment of BN particles on the thermal conductivity of BN/ETDS composites at various filler concentration. Reproduced from ref. 64, with permission of *Ceram. Int.*, copyright 2014. (b) Through-plane thermal conductivities of BN–LNP films with different filler loading. Inset is the corresponding thermal diffusivity of BN–LNP films; (c) thermal dissipation models of BN–LNP and BN/LNP composites. Reproduced from ref. 115, with permission of *Compos. Sci. Technol.*, copyright 2021. Dielectric constant (d) and dielectric loss (e) of a table fabricated with cross-linked PBZA–H as a function of frequency under different annealing temperatures at room temperature. Dielectric constant (f) and dielectric loss (g) of cross-linked PBZA–V as a function of frequency under different annealing temperatures at room temperature. Reproduced from ref. 66, with permission of *Mater. Sci. Eng., B*, copyright 2014. Photodegradation of Rhodamine B (RhB) under different irradiation conditions: (h) solar light irradiation *λ* > 300 nm, and (i) visible light *λ* > 420 nm by P25 and porous BN/TiO_2_ hybrid nanosheets (38 wt%). (j) Visible absorbance spectra of the RhB solution with the porous BN/TiO_2_ hybrid nanosheets (38 wt%) under visible light irradiation. The inset shows the photographs of the RhB solutions after different irradiation time. (k) The degradation performance of RhB of porous BN/TiO_2_ hybrid nanosheets (38 wt%) under visible light with 5 successive cycles. Reproduced from ref. 121, with permission of *Appl. Catal., B*, copyright 2017.

Kim *et al.* used a series of material advancements to successfully create thermoplastic polyurethane elastomer composites with thermally conductive fillers. Thermoplastic polyurethane/BN composites were created in their first study with optimized filler–matrix interfaces and 20% better heat conductivity (0.467 W m^−1^ K^−1^) than the original polymer.^65^ Building on this basis, further optimization of epoxy/BN composites using wetting-controlled processing showed improved particle–matrix interactions, a high cohesive energy density of the hydroxyl groups, and the highest thermal conductivity for the BN–OH/ETDS composite, 2.85 W m^−1^ K^−1^, which was 1.44 times greater than that of the pristine ETDS (Fig. 5a). On the other hand, because of the covalent connection between silane-modified BN surfaces and epoxy networks, the surface curing agent was decreased, yielding a value of 1.68 W m^−1^ K^−1^, which is 0.59 times lower than that of the BN/ETDS composite.^64^ This study offers a useful strategy for multipurpose uses of long-lasting, thermally conductive, and insulating packaging materials. Dai's research on hierarchical cellulose nanocomposites in 2021 led to advancements in the field. In order to achieve thermal conductivity (2.577 W m^−1^ K^−1^) at 50 weight percent BN loading, the team constructed dual thermal pathways through borax-crosslinked BN–lignin networks within cellulose nanofibril (CNF) matrices (Fig. 5b). This property was only 0.413 W m^−1^ K^−1^ for the pure CNF film, indicating an improvement of approximately 524% while preserving flexibility.^115^ The BN–LNP50 composite was notable for having a 30% higher thermal degradation onset (230 °C) than pure CNF and for having a 99.7° initial WCA. It also showed good hydrophobicity. The thermal dissipation models are shown in Fig. 5c, the BN–LNP/CNF composites will be widely used as multifunctional and extremely efficient TIMs in next-generation electronics. Parallel advancements in crystalline BN use resulted from Kodambaka's 2023 heteroepitaxial growth investigation. Using borazine-pyrolyzed hBN as a van der Waals substrate, the team created oriented MoS_*x*_ films with higher crystallinity than Al_2_O_3_ (0001).^116^ This provides a scalable way to manufacture 2D semiconductor heterostructures, which are required for next-generation nanoelectronics. By utilizing deposition equipment that can handle wafer-sized substrates and optimizing growth conditions, their method may be scalable.

By reacting isopropylamine with trichloroborazine (TCB) in mild circumstances, Xie *et al.* invented the simple synthesis of poly(isopropylamino)borazine (PTPiAB), a soluble and melt-processable preceramic polymer.^57^ Hexagonal boron-nitride (h-BN) with remarkable qualities, such as low density (2.03 g cm^−3^), outstanding high-temperature oxidation resistance (mass loss < 0.3% at 900 °C), and an ultra-low dielectric constant (2.48 at 10 GHz), was produced by further pyrolysis of PTPiAB. It is a strong contender for high-frequency electronic packaging because of these qualities. Building on this basis, Wang and Zhu's group used a condensation process between *B*,*B*′,*B*′′-trichloroborazine and an arylacetylene Grignard reagent to create a high-yield (>75%) borazine-containing arylacetylene resin (PBZA).^66^ In comparison to standard polyimides (*ε* > 3.0), the cured PBZA demonstrated exceptional thermal stability (Td_5_ = 427.4–496.7 °C) and excellent dielectric characteristics, with a dielectric constant (*ε*) of 2.7 and a dielectric loss of 0.002 at 0.1 Hz to 1 MHz (Fig. 5d–g). By creating a boron–nitrogen cyclic silicon aromatic alkyne resin (PBZSA) through the condensation of *B*,*B*′,*B*′′-trichloroborazine, dichlorosilane, and arylacetylene Grignard reagents, the researchers added silicon moieties to further enhance interfacial compatibility.^117^ The resultant PBZSA bridged the gap between inorganic thermal resistance and organic processability by demonstrating synergistic gains while preserving ultra-low dielectric loss. After pyrolysis at 1000 °C, the insoluble solid is converted into Si_3_N_4_ and BN amorphous structures with a ceramic yield of almost 95%. In addition to advancing materials science, our study supports global demands for quantum technologies, 5G infrastructure, and energy-efficient electronics. Ceramics made from borazine have the potential to expand the capabilities of high-frequency and high-power microelectronics by resolving issues with scalability and dependability.

Since 2017, Bonifazi's team has been at the forefront of a methodical molecular engineering approach to BN-doped materials, setting new standards for accurate doping of aromatic systems. The synthesis of borazine coronene derivatives through strategic benzene-to-borazine substitution was the first step in their groundbreaking work.^15^ For deep blue organic light-emitting diodes (OLEDs), they were able to create the first soluble hexa-*peri*-hexabenzoborazinocoronene through a rational synthesis. Spectroscopic analysis of this compound showed a blue shift in emission maxima and a widening of the HOMO–LUMO gap in comparison to all-carbon analogs. This was the first instance of BN doping being used to demonstrate programmable electronic structure modulation in graphene-like materials. Building on this basis, the group created a flexible [4 + 2] Diels–Alder cycloaddition method to create hybrid BNC polyphenylenes the same year.^118,119^ They were able to achieve tunable bandgap engineering while preserving the purity of the π-conjugation by substituting borazine rings for specific aryl units. In 2019, tri-OTF-borazine compounds were added to the approach, enabling the modular assembly of photoactive BNC hybrids.^60^ With a mass loss of 5 weight percent at 203 °C and a loss of around 30 weight percent at 351 °C, the polymer shows good thermal stability. Through ethynylborazine cycloaddition, they made history in 2022 by introducing the first borazine-doped organogel.^38^ This material proved useful as a solid-state electrolyte (SSE) matrix in lithium-ion batteries due to its high thermal stability (decomposition beginning between 300 and 450 °C) and ionic conductivity of 1.51 × 10^−5^ S cm^−1^ with a Li transfer number of roughly 0.2. In 2024, new 2D structures were discovered by surface-mediated borazine chemistry on Ag (111).^120^ Borazine's potential for creating quantum materials with topological characteristics was demonstrated by the creation of chiral Kagomé lattices with Dirac cone-like electronic structures through controlled dehalogenation/dehydrogenation. This research substantially transforms borazine chemistry from a specialized synthetic challenge to a platform technology for next-generation materials. As the discipline advances toward applications in quantum computing, flexible electronics, and beyond-energy storage, Bonifazi's team's molecular engineering concepts serve as a road map and source of inspiration for the larger materials science community.

By creating porous BN/TiO_2_ nanosheets with interfacial B–O–Ti covalent linkages, Lei and associates created hybrid photocatalysis.^121^ The team reduced the effective bandgap by 0.18 eV in comparison to pure TiO_2_ by attaching TiO_2_ nanoparticles to the boron-terminated edges of porous BN. Porous BN/TiO_2_ hybrid nanosheets with the highest photocatalytic activity (38 wt%) achieved a degradation percentage of up to 99% under solar light irradiation (*λ* > 300 nm) and visible light (*λ* > 420 nm). This is significantly higher than that of P25 (∼60%), as illustrated in Fig. 5h and i. As seen in Fig. 5j, the photos of the RhB solutions demonstrate the color shift from pink to clear with longer exposure times to visible light, further verifying the full photodegradation of RhB. Furthermore, the porous BN/TiO_2_ hybrid nanosheets maintain strong visible light photocatalytic activity (97%) and show outstanding cycling stability for up to five cycles (Fig. 5k). These findings offer fresh perspectives on the development of sophisticated hybrid photocatalysts with active chemical binding species for use in water splitting, photoelectrochemical conversion, and environmental protection. A semi-synthetic pathway to B_3_N_3_-doped few-layer graphene (BNG) was developed by Qi *et al.* in conjunction with this work by the controlled pyrolysis of B_3_N_3_ acetylene precursors.^122^ In comparison to undoped graphene, the resultant materials showed an increase in oxygen reduction reaction (ORR) activity and an adjustable bandgap (1.8–2.4 eV). Charge redistribution at the B–N–C interfaces was discovered by X-ray absorption near-edge structure (XANES) analysis, resulting in localized electron-deficient areas that improve catalytic site accessibility. These works are rethinking hybrid material design criteria, shifting away from trial-and-error composite mixing and toward atomic precision interfacial engineering. As global demand for green chemistry and sustainable energy grows, rationally designed BN–carbon–metal oxide systems may hold the key to next-generation catalytic technologies.

### 5.3 Absorbent material

**Fig. 6:**
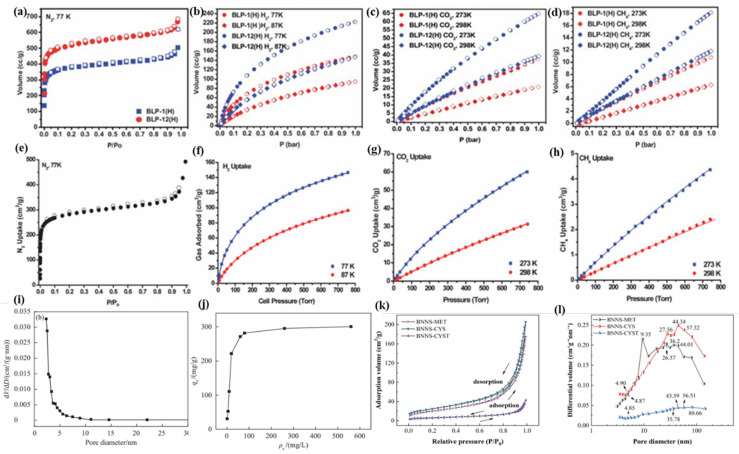
(a)–(d) Gas uptake isotherms for BLP-1(H) and BLP-12(H); adsorption (filled) and desorption (empty). Reproduced from ref. 63, with permission of *Polym. Chem.*, copyright 2011. Gas uptake isotherms for BLP-10(Cl), N_2_ (e), H_2_ (f), CO_2_ (g), and CH_4_ (h); sorption (filled) and desorption (empty). Reproduced from ref. 67, with permission of *J. Mater. Chem.*, copyright 2012. (i) Nitrogen adsorption-pore size distribution; (j) experimental results of Cu^2+^ adsorption equilibrium. Reproduced from ref. 56, with permission of *China Surfactant Deterg. Cosmet.*, copyright 2024. (k) N_2_ adsorption and desorption isotherms for BNNS-SAAs, (l) pore size distribution curves for BNNS-SAAs calculated by the BJH method. Reproduced from ref. 123, with permission of *Chem. Eng. J.*, copyright 2024.

By utilizing their distinct electron-deficient boron centers and adjustable pore topologies, borazine-based porous polymers are becoming a paradigm shift in the field of adsorbents. By creating borazine-linked polymers (BLPs) by condensing arylamines with boron trihalides, El-Kaderi's group was a pioneer in this field.^101^ The surface areas of the optimized BLP version ranged from 503 to 1364 m^2^ g^−1^. Amazing gas uptake capabilities are made possible by this nanostructure: at the beginning, the temperature was 77 K (1 bar), and the hydrogen weight percentage was 1.3 wt%. A second highly porous borazine-linked polymer (BLP) was then synthesized and gas-absorbed by the researchers.^63^ At 1.0 bar, BLPs have isosteric heats of adsorption of 6.0 and 25.2 kJ mol^−1^, respectively, and can hold significant amounts of H_2_ (1.93 wt%) and CO_2_ (12.8 wt%) at 77 K and 273 K, respectively (Fig. 6a–d). They also have high surface areas, up to 2866 m^2^ g^−1^. According to the hydrogen sorption studies, BLPs exhibit a slightly greater hydrogen isosteric heat of adsorption and have large hydrogen storage capacities at low pressure. A year later, they released a paper that looked into a number of borazine-linked polymers using the straightforward synthesis method of arylamine thermolysis with boron trihalides.^55,67^ According to the gas storage research, BLPs have significant isosteric temperatures of adsorption and sufficient amounts of H_2_ and CO_2_ gas storage capacity. BLPs are a good choice for gas storage and separation applications because of their high level of efficacy in CO_2_/CH_4_ selectivity investigations, as shown in Fig. 6e–h. Because of their high surface areas, the resulting polymers are attractive for use in gas sorption and separation. A variety of halogen and aryl building blocks can be carefully added to modify these characteristics. With the use of creative synthetic techniques that allow for exact control over porosity and surface chemistry, the boundaries of functionalized boron nitride (BN) materials are quickly expanding. Bonifazi's group used tricarboxylic hexaarylborazine ligands to successfully create a novel 3D BN–metal organic framework (MOF). Since the framework can be structurally altered through organic functionalization, as previously described, this accomplishment represents a major breakthrough in the field.^36^ With a BET surface area of 1091 m^2^ g^−1^, this architecture demonstrated a remarkable capacity to extract CO_2_ (3.31 mmol g^−1^ at 273 K, 1 bar). In 2024, Long *et al.* developed a precursor-mediated method for hierarchical BN by pyrolyzing tris(methylamino)borazine under regulated conditions.^56^ Porous boron nitride has a surface area of 558.07 m^2^ g^−1^, a single-point average pore radius of 2.03 nm, and a mesoporous structure with pore sizes of about 2.5 nm (Fig. 6i). With an adsorption rate of over 90%, the adsorption of porous boron nitride on metal ions was found to be complete; however, the amount of activated carbon that was adsorbed was less than 30 mg g^−1^, and the adsorption rate was less than 50%. In accordance with the actual value of 296.50 mg g^−1^, the maximum adsorption of Cu^2+^ by porous boron nitride was determined to be 295.62 mg g^−1^, suggesting that the adsorption is homogeneous and monolayer (Fig. 6j). The drawbacks of inorganic preparation—where the components are irregularly distributed, prone to moisture absorption, and susceptible to pulverization—are addressed by using an organic precursor technique to create boron nitride.

Recently, sulfur-functionalized BN nanosheets (BNNS) have become a crucial component of the BN applications that Zhou's team has extended to include sustainable agriculture.^123^ The specific surface area (SSA) and pore size distribution of BN-based materials were determined using the BET and BJH methods; the findings are shown in Fig. 6k and l. It was clear that the pore distributions of BNNS-MET and BNNS-CYS were similar to those of BNNS, with the largest pore sizes being 9.35 nm and 44.34 nm, respectively. With a strong adhesion capacity, BNNS-CYST was discovered to be the largest main pore due to CYST-induced pore blockage. Additionally, when compared to pure TTO, BNNS-CYST-TTO showed a significant synergistic antibacterial action, as shown by an increased inhibition zone diameter of 26.89% against Ralstonia solanacearum. The minimal inhibitory concentration decreased from 1.8 mg mL^−1^ to 0.6 mg mL^−1^ at the same time. By providing extra sulfur nutrients, BNNS's exceptional wettability and stickiness on seed and leaf surfaces aided in seed germination. It has been demonstrated that the active ingredient's increased adhesion intensifies its antibacterial action, which may be used to prevent and manage plant diseases like bacterial wilt.

Table 1 shows a comparison of the above-mentioned organic borazine-based adsorbent polymer materials and boron nitride-based adsorbent polymer materials, including boron nitride nanosheet materials, hexagonal boron nitride nanosheet materials, and the like. The gas adsorption properties of these materials are also compared with those of representative other porous materials. It can be seen that the organic borazine-based adsorbent polymer materials have good gas adsorption properties comparable to those of boron nitride-based adsorbent polymer materials. Compared with these representative porous materials, the organic borazine-based adsorbent polymer materials have much room for improvement and optimization. A superposition of features results from the assembly and combining of porous materials with superior adsorption capabilities with borazine or hexagonal boron nitride. In 2021, Alfuhaidi and Abdel functionalization of CB-doped h-BN on one and two sides can provide high H_2_ storage performance for fuel cells.^124^ By improving the structure with C substitution of boron and nitrogen atoms in the h-BN nanosheets, they successfully increased the binding energy of the decorated metals. As an alternative to costly catalysts, helpful instructions are offered for the design and enhancement of catalyst architectures for BN sheet-based electrocatalysts. Zhu *et al.* reported in 2024 that multilayer h-BN was assembled with MOF-NUS-8 to create hybrid NUS-8/BN with a sandwich structure.^136^ The adsorption capacity of NUS-8/BN for H_2_ was significantly enhanced, and it was 1.7 times greater than that of h-BN nanosheets, because of the richer exposed binding sites and partial ionic bonding characteristics of BN. Borazine polymers are now at the forefront of sustainable materials science thanks to these technological positions. The apparent achievements in energy, the environment, and agriculture highlight BN's potential to overcome traditional material constraints and usher in a new era of “smart” porous materials designed at the atomic level for macroscopic effects. These materials' stability, tunability, and biocompatibility together have the potential to solve important global issues. In order to unleash next-generation smart adsorbents for the hydrogen economy and circular carbon sectors, stability challenges must be resolved while utilizing computational design methods.

**Table 1:** Comparison of gas adsorption properties of adsorbent polymeric materials based on organic borazine or boron nitride and other porous materials

Polymers	Surface areas (m^2^ g^−1^)	H_2_ uptake	CO_2_ uptake	Isosteric heat of adsorption (kJ mol^−1^)	Ref.
BLP	503–1364	1.3 wt%	—	7.1 (77 K/1 bar)	101
BLP-12 (H)	2866	1.93 wt%	12.8 wt%	6 (77 K/1 bar)	63
				25.2 (273 K/1 bar)	
BLP-12 (Cl)	1174–1569	0.68–1.75 wt%	51–141 mg g^−1^	7.06–7.65 (77 K/1 bar)	55
				22.2–31.7 (273 K/1 bar)	
BLP-10 (Cl)	1308	1.3 wt%	—	7.46 (77 K/1 bar)	67
				28.3 (273 K/1 bar)	
BNPB-1100	1488	1.6–2.3 wt%	—	—	130
BNMSs	1900	1.65–2.57 wt%	—	—	131
BN-600	960	1.01 wt%	—	12 (77 K/1 bar)	132
h-BN nanosheets	2800–4800	0.73 wt%	—	6.2 (77 K/1 bar)	135 and 136
NUS-8/BN-3	1232	1.23 wt%	—	8.1 (77 K/1 bar)	136
2D COFs	970–4620	14.8–39.2 mg g^−1^	230–1010 mg g^−1^	6–7 (77 K/1 bar)	125
BPL carbon	1500	25.5 mg g^−1^	370 mg g^−1^	8 (77 K/1 bar)	125
3D COFs (PAF-1)	7100	10.7 wt%	1300 mg g^−1^	4.6 (77 K/1 bar)	129
MOF-5 (IRMOF-1)	4400	76 mg g^−1^	970 mg g^−1^	4.8 (77 K/1 bar)	126–128
MOF-177	5640	75.2 mg g^−1^	1490 mg g^−1^	4.4 (77 K/1 bar)	126, 133 and 134

### 5.4 Aerospace and military materials

**Fig. 7:**
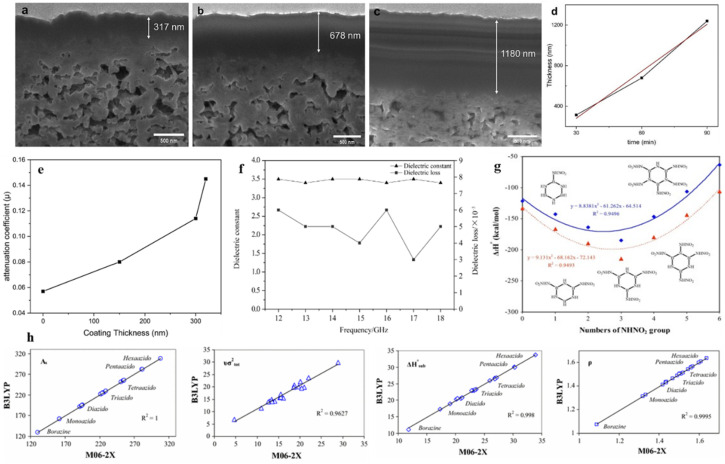
Focused ion beam (FIB) cross-sectional images of CNT mat-BN composites with various coating times: (a) 30 min; (b) 60 min; (c) 90 min. (d) Thickness of BN coating layer as a function of time. (e) Neutron shielding coefficient of raw CNT mat and CNT mat-BN composites with various coating thickness. Reproduced from ref. 143, with permission of *Surf. Coat. Technol.*, copyright 2024. (f) Dielectric properties of 2.5D SiO_2f_/BN. Reproduced from ref. 141, with permission of *Composite Materials: Science and Engineering*, copyright 2020. (g) Influence of the numbers of –NHNO_2_ group on the Δ_f_*H*° (g) (solid line) and Δ_f_*H*° (s) (dotted line) data for more stable isomers of nitraminoborazines. Reproduced from ref. 145, with permission of *Comput. Mater. Sci.*, copyright 2015. (h) Relationship between M06-2X and B3LYP methods for prediction of the molecular surface properties, crystal density (*ρ*) and enthalpy of sublimation of azidoborazines. Reproduced from ref. 146, with permission of *J. Iran. Chem. Soc.*, copyright 2015.

Wave-transparent materials are required for radomes of various types of vehicles, such as hypersonic vehicles. Materials researchers around the world have developed various wave-transparent materials to prepare radomes to meet the working requirements. Cao's team has prepared wave-transparent composites made of boron nitride ceramic matrix reinforced with silicon nitride fiber and borazine as reinforcing body and ceramic precursor, respectively.^68,137,138^ The material not only exhibits strong high-temperature mechanical properties in terms of high-temperature resistance, but also has good dielectric properties and thermal stability. It has potential applications in aerospace, such as the manufacture of long-term temperature resistant radomes (antenna windows). Subsequently, the team of Zhang *et al.* carried out a study on the preparation process and properties of nitride fiber-reinforced boron nitride ceramic-based wave-transparent composites using borazine as a precursor for the boron nitride matrix.^139,140^ The study found that ceramicisation could be achieved essentially by cracking at 800 °C. High-purity, high-density and isotropic BN ceramics were prepared by a new method combining mould sintering and precursor impregnation and cracking processes using polyborazine powders as raw materials. The thermal conductivity, coefficient of thermal expansion, dielectric constant and loss angle tangent of the BN ceramics gradually increased with the increase of the preparation temperature. It is also expected to be used as a wave-transparent material in aerospace for aircraft or missile radomes. In 2020, Gao *et al.* prepared 2.5D quartz fiber reinforced boron nitride ceramic matrix composites using borazine as the boron nitride ceramic precursor and 2.5D quartz fiber precursor as the reinforcement.^141^Fig. 7f shows that the dielectric loss is less than 10 × 10^−3^ and that the dielectric constant of 2.5D SiO_2f_/BN composites at 12–18 GHz is maintained at 3.4–3.5. 2.5D SiO_2f_/BN composites have excellent dielectric properties and can be used as wave-transparent materials for radomes. The dielectric properties of wave-transparent materials for radomes are typically expressed by the dielectric constant *ε* and the tangent of the loss angle tan *δ*. The excellent wave-transparent materials have a dielectric constant *ε* of only 1–4 and a tan *δ* of (1–10) × 10^−3^.^142^ It was shown that the composites had a dense outer layer and a loose and porous inner layer, with excellent dielectric properties, room temperature mechanical properties and high temperature mechanical properties, and could be used as radome wave-transparent materials.

The authors of the study by Kim *et al.* (2024) used borazine to coat BN onto carbon nanotube (CNT) mats using chemical vapour deposition (CVD) to create carbon–boron nitride (C–BN) composites.^143^ SEM investigation (Fig. 7a–d) showed that surface holes gradually filled as BN thickness increased (317–1180 nm). The trend line's gradient of 14.3 nm min^−1^ indicates that adjusting the growth period may be able to modify the material's thickness.^144^ The large-area CNT mat's growth rate of 14.3 nm min^−1^ throughout its whole surface is noticeably faster than the synthesis employing heterogeneous precursors documented in earlier research. As shown in Fig. 7e, the neutron shielding factor was increased to 0.145 mm^−1^, which is 2.5 times greater than that of uncoated CNTs, by a 320 nm BN coating, which accounted for 0.2% of the overall thickness. This dense BN coating has been found to improve oxidation resistance, tensile strength (64–111 MPa *vs.* 50 MPa for raw CNTs) and neutron attenuation. These findings establish these composites as key materials for aerospace radiation shielding and extreme environment applications.

Keshavarz *et al.* conducted theoretical calculations to evaluate borazine-derived energetic compounds with nitroamino groups. These simulations anticipated that these materials will have better detonation performance than ordinary organic nitroamines.^145^ Theoretical calculations predict the negative values of −184.7 to −9.4 kcal mol^−1^ for Δ_f_*H*° (g) and −204.8 to −30.3 kcal mol^−1^ for Δ_f_*H*° (s) of nitraminoborazines. The stability of these species decreases when the number of substituents is more than three (Fig. 7g). This research suggests that nitroborazine compounds have good thermodynamic stability, and that reducing the negative condensed phase may improve detonation performance. The molecular structure of novel high-energy materials was described, including borazine and modified –NHNO_2_ groups, as well as –N_3_ groups. These groupings outperform traditional organic nitramines, such as tetryl, DNNT, DANT, RDX, and HMX. The detonation velocity of these compounds is high, approaching 10 000 m s^−1^. They also discovered that the crystal density and enthalpy of sublimation of the azidoborazines both dramatically increase with the number of azide substituents, as shown in Fig. 7h.^146,147^*B*-Substituted azidoborazines are more stable than *N*-substituted ones, despite the fact that their stability is greatly diminished. Additionally, the *B*-substituted triazaborine and diazoborazine compounds have higher crystal densities than the *N*-substituted ones. Azidoborazine has better detonation qualities than nitroborazine and nitroaminoborazine because its condensed phase enthalpy of formation is more positive. Strong-energy azide substituents can be added to nitroborazine to change its moderate detonation characteristics and strong thermodynamic stability. Therefore, strong-energy azide substituents can be added to nitroborazine to change its moderate detonation characteristics and strong thermodynamic stability.

This development puts BN ceramic composites in a position to facilitate the development of next-generation aircraft systems. An example of materials-by-design is the borazine paradigm, which shows how an obscure inorganic ring may be transformed into a platform for advancements in sustainability, energy, and defense. Future research initiatives must focus on cost-cutting measures and lifecycle management techniques while utilizing the potential of newly developed computational materials design tools. Convergent research that bridges systems engineering, computational modeling, and synthetic chemistry is necessary to realize its full potential.

### 5.5 Catalytic material

**Fig. 8:**
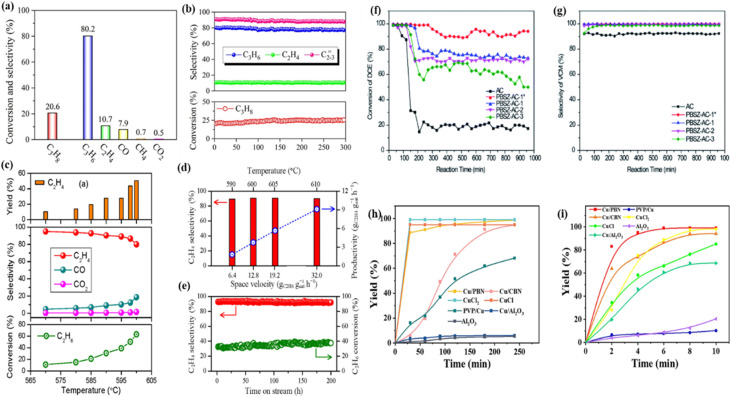
(a) Propane conversion and product selectivity; (b) long-term stability test. Reproduced from ref. 149, with permission of *ChemCatChem*, copyright 2017. (c) Dependence of ethane conversion, ethylene selectivity and yield on the reaction temperature; (d) effect of space velocity and reaction temperature on the productivity of ethylene; (e) long-term stability test at 590 °C. Reproduced from ref. 151, with permission of *Chin. J. Catal.*, copyright 2017. (f) The conversion of 1,2-DCE over the B,N-ACs catalysts; (g) the selectivity of VCM over the B,N-ACs catalysts. Reaction conditions: temperature = 250 °C, 0.1 MPa, LHSV (1,2-DCE) = 0.67 h^−1^. Reproduced from ref. 155, with permission of *RSC Adv.*, copyright 2021. (h) The yields of dehydrogenation of hydrazobenzenes with different catalysts. (i) The yields of reduction of azobenzene with different catalysts. General reaction conditions: substrate 1 (0.05 mmol), catalyst (5 mg), solvent: methanol, ambient temperature. The Cu content of all Cu-containing catalysts is 5 mg × 4.34%. Reproduced from ref. 156, with permission of *J. Catal.*, copyright 2023.

Coordination polymers based on borazine have shown promise as catalysts in a range of chemical processes, such as polymerization and hydrogenation. The potential of boron nitride nanotubes (BNNTs) and hexagonal boron nitride (h-BN) synthesized from borazine precursors for propane oxidative dehydrogenation (ODH) was initially shown by Grant *et al.* in 2016. They achieved 91% propylene selectivity at 14% conversion.^148^ This discovery paved the way for Lu's later creation of edge-hydroxylated BN (OH–BN) catalysts,^149^ which proved the new catalyst's effectiveness by achieving 80.2% propylene selectivity at 20.6% propane conversion with a negligible CO_2_ byproduct (0.5%) (Fig. 8a). As show in Fig. 8b, the operation of a 300 h test at 530 °C demonstrated the extraordinary stability of the BNOH catalyst in the ODH of propane, with no notable changes in conversion and product selectivity occurring, indicating the possibility for industrial application. Under reaction circumstances, pure boron nitride needs time to activate because it is catalytically inert.^150^ A 300 hours test with consistent conversion and product selectivity demonstrated remarkable stability. It has been shown that boron nitride effectively catalyzes the oxidative dehydrogenation of propane with a high selectivity toward propylene and a greatly reduced CO_2_ emission when activated by steam to produce B–OH groups at the edges. In addition to opening up a new avenue for study in the selective activation of alkanes' C–H bonds, this innovative, metal-free catalyst system has great potential as a catalyst for the industrial ODH process. Further validation of this mechanism was obtained in ethane ODH, where OH–BN achieved 95% ethylene selectivity at 11% conversion (CO_2_: 0.4%).^151^ It is noteworthy that even at a high conversion level of 63%, the ethylene selectivity retained at 80% is competitive with the energy-demanding industrialized steam cracking route (Fig. 8c). As demonstrated in Fig. 8d, an unparalleled ethylene productivity (9.1 g_C_2_H_4__ g_cat_^−1^ h^−1^) with >90% selectivity was attained over the BNOH catalyst by modulating the reaction conditions. The observed productivity levels significantly exceed the critical value of 1 g_C_2_H_4__ g_cat_^−1^ h^−1^, which would be commercially viable.^152^ Stable conversion and product selectivity were demonstrated over a 200 hours period, indicating excellent catalytic stability (Fig. 8e). The proven 200 hours stability is only the beginning of BN catalysts' potential applications; with continuous research, there is a significant likelihood that they may become the “enzyme analogs” of industrial catalysis, ushering in an era of precise hydrocarbon processing.

Using metal-free boron nitride (BN) nanosheets made from borazine precursors, Su *et al.* made significant progress in ethane oxidative dehydrogenation (ODH).^153^ With 100% ethylene selectivity at 10% ethane conversion and 60% selectivity even at 78% ethane conversion, the high-surface-area BN nanosheets (s-BN) demonstrated remarkable catalytic activity. They also showed stability for 400 hours at 575 °C, which is comparable to the performance of the most advanced platinum–selenium catalysts. *Operando* investigations showed that the reaction mechanism is driven by B–O(H) edge sites that are dynamically replenished *via* O_2_-assisted hydrogen abstraction. Through this work, BN has been able to use its tunable edge chemistry and intrinsic thermal stability, leading to a better understanding of the ODH reaction of alkanes. It is anticipated that these findings will serve as a catalyst for the actual production of olefins from paraffins. Building on this, Han *et al.* used a solid-phase synthesis based on borazine to create porous boron nitride microtubes (BNMTs).^154^ The distinctive tubular structure has a lot of B–O and B–OH species. At 600 °C, this architecture produced 72% ethylene selectivity and 45% ethane conversion. Compared to commercial BN catalysts (20% conversion with 80% selectivity), this performance is doubled. Building two-dimensional structures is made easier by the co-planarity of B_3_N_3_, the acetylene group, and the benzene ring, as well as the electronegativity of borazine (B_3_N_3_). The microtubes' hierarchical porosity and optimized electrical interactions at defect-rich edges are responsible for the increased activity, which makes C–H bond activation more effective. Concurrently, the Qi team pioneered B_3_N_3_-doped activated carbons (B,N-ACs) for sustainable dehydrochlorination of 1,2-dichloroethane (1,2-DCE).^155^ It has been demonstrated that incorporating the planar B_3_N_3_ units of borazine into carbon matrices leads to a 92% 1,2-DCE conversion and >99.9% vinyl chloride selectivity at 250 °C (Fig. 8f and g). This result outperforms the existing catalysts in industry benchmarks. This study's environmentally friendly and metal-free boron and nitrogen co-doped catalyst has important ramifications for future studies on the environmentally friendly and sustainable industrial method of preparing vinyl chloride utilizing boron and nitrogen co-doped carbon catalysts. If both the dehydrogenation of hydrazobenzene and the hydrogenation of azobenzenes can be accomplished under mild reaction conditions, the hydrazobenzene/azobenzene couple has been recognized as a possible hydrogen storage system.

Wang *et al.* were the first to create a sustainable catalytic system in 2023 that used porous boron nitride (PBN)-supported copper nanoparticles (Cu/PBN) for reversible azobenzene–hydrazobenzene interconversion under very mild circumstances.^156^ The Cu/PBN catalyst outperformed traditional Al_2_O_3_-supported catalysts by 90% in both oxidative dehydrogenation of hydrazobenzenes and transfer hydrogenation of azobenzenes (Fig. 8h and i). The effectiveness of the technique can be attributed to PBN's hierarchical pore structure, which allowed for substrate diffusion while stabilizing Cu clusters against sintering. The system's environmental friendliness was proved during solar-powered outdoor operation, with molecular hydrogen byproducts efficiently separated using gas-permeable BN membranes. In 2024, Hussain *et al.* developed magnetic MoO_3_–BN–Fe_2_O_3_ nanocomposites for ultrasonic-assisted oxidative desulfurization, expanding on the adaptability of BN.^157^ The elimination of dibenzothiophene (DBT) increased significantly from 42% to 99.6% in model fuels when the MoO_3_ dose was increased from 5% to 10%. *Operando* FTIR identified two mechanisms: DBT is oxidized by the Mo^6+^ sites to DBTO_2_, which then adsorbs on the electron-deficient boron centers of BN. Additionally, sonic oxidative adsorptive desulfurization of Iranian fuel oil was investigated using the most active catalyst in the desulfurization of model fuel (10% MoO_3_–BN–Fe_2_O_3_), with full and specific sulfuric component removal being noted.

Boron nitride could efficiently catalyze the oxidative dehydrogenation of propane to propylene with impressive selectivity, and with only negligible CO_2_ formation. It is important to note that the long-standing issue of controlling olefin selectivity during oxidative dehydrogenation is resolved by this novel catalyst system. The use of some typical catalysts produces large amounts of unwanted CO_2_ due to excess oxidation reactions, such as those listed in Table 2, in which *Meso*-NiMoO_4_ produces about 30% CO_2_ and the conversion of Pt_8–10_/SnO/Al_2_O_3_ CO_2_ is at 18%.^161,162^ As illustrated in Table 2, the borazine-based catalysts demonstrate comparable or superior olefin conversion and selectivity to certain representative catalysts. As a cross reference, the selective oxidation of *n*-butane and isobutane, which is catalyzed by hexagonal boron nitride^158^ and the sophisticated catalyst representation carbon nanotubes (CNTs)^159^ and supported V catalysts,^160^ is also included. This development from basic BN nanosheets to tailored microtubes and hybrid carbons highlights how versatile borazine chemistry is in creating metal-free, next-generation catalysts for green chemical synthesis and hydrocarbon valorization. This development from basic azobenzene cycle to industrial desulfurization applications highlights BN's revolutionary potential in green catalysis, connecting scalable environmental remediation technologies with molecular precision. An excellent illustration of how materials chemistry may be used to solve persistent industrial problems is this catalytic revolution. With improved selectivity, lower emissions, and metal-free sustainability, borazine-derived BN catalysts offer a strong triple-win option in the face of growing pressure on the petrochemical sector to cut carbon emissions.

**Table 2:** Comparison of several oxidative dehydrogenations of different alkanes based on organic borazine and representative catalysts

Oxidative dehydrogenation (ODH)	Catalyst	Conversion (%)	Stability	Selectivity (%)	Productivity	Ref.
Propane	BNOH	20.6	530 °C, 300 h	80.2	6.8 g_olefin_ g_cat_^−1^ h^−1^	149
	h-BN	14	—	79	∼1 g_olefin_ g_cat_^−1^ h^−1^	148 and 150
	BNNT	11	—	75	4 g_olefin_ g_cat_^−1^ h^−1^	148 and 150
	*Meso*-NiMoO_4_	14.1	—	72	10.2%	161
	Pt_8–10_/SnO/Al_2_O_3_	25 ± 4	500 °C, 30 h	64–83.7	84%	162
Ethane	BNOH	11	200 h	95	9.1 g_olefin_ g_cat_^−1^ h^−1^	151
	s-BN	9.2	575 °C, 550 h	100	—	153
	BNMTs	45	—	72	—	154
	B,N-ACs	92	—	99.9	—	155
*n*-Butane	h-BN	7.2	—	76.2	—	158
	CNT	5	—	70	—	159
Isobutane	h-BN	6.2	—	75.4	—	158
	Cr/Al_2_O_3_	5	—	64	—	160

## 6. Conclusion and outlook

As an emerging functional polymer material with the potential to replace toxic flame retardants and reduce reliance on precious metal catalysts, borazine-based polymer materials have made remarkable advances in areas such as synthesis technology, performance exploration, and application development. The addition of borazine rings not only increases the materials' thermal stability and oxidation resistance, but also provides a number of features such as low dielectric, gas adsorption, catalytic, and photovoltaic functions. These unique chemical characteristics and designable architectures pave the way for a more sustainable green chemical industry and materials applications. At present, various types of borazine-based polymer materials have been successfully developed, such as borazine-doped conjugated polymers, borazine-based ceramic precursors, and porous borazine-conjugated polymers, *etc.*, which have shown great potential for applications in high-temperature flame retardants, aerospace, microelectronics, and energy and environment.^163–166^

Although significant progress has been made, challenges remain in achieving precise structural control, long-term stability and performance optimization of borazine-based materials. The complexity of the synthesis of borazine monomers and polymers and the high reactivity of borazines result in the need for precise control of conditions during the functionalization process; some of the dynamically bound materials are prone to degradation in extreme environments, *e.g.* strong acids and bases, resulting in failure to ensure long-term stability and the high synthesis cost of high-purity borazines limits industrial applications, requiring optimization of properties and hence cost reduction to achieve large-scale production. By replacing persistent pollutants (like brominated flame retardants) with borazine's natural flame retardancy and environmental inertness, and by creatively creating hydrolyzable dynamic covalent bonds or chemically recyclable skeletons to accomplish closed-loop recycling of the material.^167–170^

Future research directions may include the development of new borazine monomers, the exploration of advanced aggregation methods and the expansion of applications in areas such as catalysis, sensing and energy storage. To enable borazine-based materials to have improved properties and a wider range of applications through the development of new green and efficient synthesis and functionalization methods.^171,172^ Meanwhile, by merging green functionalization methods (*e.g.*, aqueous phase modification, metal-free catalysis), it will broaden their applications in environmental remediation, renewable energy storage, and low-consumption separation membranes.^173–177^ Explore new application areas; the unique properties of borazine-based polymers suggest that they have the potential to be used in emerging areas such as flexible electronics, artificial muscles, sensors and biomedical devices.^178–183^ while incorporating computational chemistry (*e.g.* DFT simulations) to guide the rational design of materials.^184^ In conclusion, borazine-based functional polymers offer a promising platform for the design of novel materials with tunable structures and high performance.

## Conflicts of interest

There are no conflicts to declare.

## Data Availability

No primary research results, software or code have been included and no new data were generated or analysed as part of this review.
